# Bloom helicase contributes to successful crossover formation with both catalytic and structural roles in *Caenorhabditis elegans* meiosis

**DOI:** 10.1093/nar/gkaf1030

**Published:** 2025-10-22

**Authors:** Sowmya Sivakumar Geetha, Ivana Čavka, Maria Rosaria Dello Stritto, Angela Graf, Tomas Macha, Hannah Krakolinig, Simone Köhler, Verena Jantsch

**Affiliations:** Max Perutz Labs, Department of Chromosome Biology, University of Vienna, Vienna BioCenter, 1030 Vienna, Austria; Vienna BioCenter PhD Program, a Doctoral School of the University of Vienna and the Medical University of Vienna, 1030 Vienna, Austria; The European Molecular Biology Laboratory, Cell Biology and Biophysics Unit, 69117 Heidelberg, Germany; Collaboration for joint PhD degree between EMBL and Heidelberg University, Faculty of Biosciences, 69117 Heidelberg, Germany; Max Perutz Labs, Department of Chromosome Biology, University of Vienna, Vienna BioCenter, 1030 Vienna, Austria; Max Perutz Labs, Department of Chromosome Biology, University of Vienna, Vienna BioCenter, 1030 Vienna, Austria; Max Perutz Labs, Department of Chromosome Biology, University of Vienna, Vienna BioCenter, 1030 Vienna, Austria; Max Perutz Labs, Department of Chromosome Biology, University of Vienna, Vienna BioCenter, 1030 Vienna, Austria; The European Molecular Biology Laboratory, Cell Biology and Biophysics Unit, 69117 Heidelberg, Germany; Max Perutz Labs, Department of Chromosome Biology, University of Vienna, Vienna BioCenter, 1030 Vienna, Austria

## Abstract

Crossover (CO)-biased repair of meiotic DNA double-strand breaks is essential for proper chromosome segregation. However, only a subset of programmed induced DSBs is repaired as COs, while the rest is processed into non-COs. The **B**loom-**T**opoisomerase 3–**R**MI1/2 complex is well documented to disassemble joint recombination intermediates into non-COs, but its pro-CO activities are less well understood. Here, we investigate how the pro-CO activities of the *Caenorhabditis elegans* Bloom helicase ortholog HIM-6 contribute to meiotic recombination by studying a catalytically inactive mutant. We show that HIM-6 helicase activity is required to provide a continuous flux of substrates for CO formation, probably via its unwinding activities, and that a structural role is sufficient to channel intermediates into the preferred pathway to generate correctly positioned COs. We provide evidence that the catalytic activity of Bloom helicase influences the geometry of the joint DNA molecules (double Holliday junctions (dHJ)). Localization of the signal for the dHJ-stabilizing complex MutSγ was more restricted, and epistasis experiments suggest that an altered geometry impedes the efficient processing of joint DNA molecules to generate CO-biased cleavage products.

## Graphical Abstract

**Graphical Abstract:**
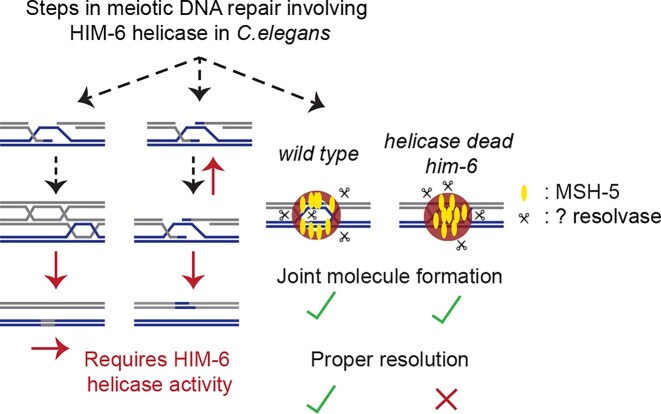

## Introduction

In meiosis, programmed DNA double-strand breaks (DSBs) entail the formation of a physical connection between the two homologous chromosomes from the parents. To this end, DSB repair by homologous recombination generates crossovers (COs) and these, together with cohesion, ensure faithful homolog segregation during the first meiotic division [1].

DSB formation is followed by resection to produce single-stranded 3′ DNA overhangs that are stabilized by the single strand binding protein replication protein A (RPA), which is then replaced by the recombinase RAD-51. The RAD-51-coated overhangs invade homologous DNA sequences and are extended by DNA synthesis, followed by strand displacement to form D-loops. D-loops can be withdrawn from the heteroduplex and re-anneal with the original chromatid to form a noncrossover (NCO); alternatively, second-end capture can generate joint DNA molecules that can mature into a CO. An outcome of CO recombination is that chromatid portions are reciprocally exchanged [2]. The formation of at least one obligate CO is ensured by the induction of an excess of DSBs and control mechanisms that fine-tune the balance between CO and NCO formation. Pro-CO factors including the MutSγ complex-like MSH-4/5, cyclin-related protein COSA-1/CNTD1, the ubiquitin/sumo ligases HEI10, RNF212, or ZHP-3/4 support differentiation into a CO product [3, 4]. Joint DNA molecules such as double Holliday junctions (dHJs) are then cleaved by nucleases (resolvases). In *Caenorhabditis elegans*, the nucleases comprise SLX-1/MUS-81 and XPF-1 together with the Bloom helicase HIM-6, and they operate in two redundant pathways. HIM-18, the worm homolog of Slx4, serves as a scaffolding protein for both pathways [5–8].

Alternatively, joint DNA molecules can be dismantled by the BTR complex (comprising the Bloom helicase, topoisomerase 3, and RMI1/RMI2 scaffold proteins) [9–11]. *In vitro* reconstitution and structural studies suggest that the Bloom helicase moves the junctions of the joint molecules toward each other until a single hemicatenane remains, which is then removed by topoisomerase 3 together with the RMI1/RMI2 scaffold proteins via single-stranded DNA cleavage, DNA strand passage, and nick re-sealing through tyrosine transesterification [12, 13]. In the context of joint molecule dissolution, the Bloom helicase catalyzes the convergent branch migration of two Holliday junctions by providing the ATP-dependent motor activity to unwind the DNA molecules [9]. Alternative models explain dHJ branch migration by coordinated action of the Bloom helicase and topoisomerase 3 [14]. *In vitro* studies of the yeast BTR complex suggest that the structural activity of the yeast Bloom helicase Sgs1 contributes to decatenation by modulating strand passage [13]. In addition, direct interaction between Sgs1 and topoisomerase 3 was found to be crucial for somatic growth and meiosis, even in the absence of Sgs1 catalytic activity [15], underscoring an important structural role for the helicase. At putative CO sites in *C. elegans* meiosis, the Bloom ortholog HIM-6 has been found in doublets, possibly flanking a dHJ-like structure [16]. The BTR complex also mediates D-loop reversion [17, 18], and this activity has also been reconstituted *in vitro* [19]. Components of the BTR complex can also reverse strand invasion events into homeologous sequences during mitosis and meiosis and, thus, prevent the formation of chromosome translocations or rearrangements [20–25]. In *Drosophila* Bloom helicase mutants, loss of the distinctive CO pattern leads to chromosome nondisjunction [26]. In summary, in meiosis the BTR complex is likely to participate in multiple steps during meiotic recombination in many organisms.

Analysis of *C. elegans* mutants showed that the BTR complex proteins HIM-6 (Bloom helicase), TOP-3 (topoisomerase), RMH-1/2 (the RMI1 homologs), and RMIF-2 (the RMI2 homolog) provide both CO and NCO activities, suppress heterologous recombination, and prevent CO formation at chromosome centers [5, 16, 21, 22, 27, 28]. Interestingly, in worms, the pro-CO activity is prominent, as evidenced by the presence of unconnected univalent chromosomes in diakinesis nuclei in mutant animals lacking BTR components. However, the source of the univalents remains unknown. HIM-6 has been proposed as a structural support to orient the dHJ during resolution by the nuclease XPF-1 to support the favored CO outcome [5, 7, 8].

Our analysis of *C. elegans* germlines of a catalytically inactive Bloom helicase (**h**elicase **d**ead, *hd him-6*) mutant revealed that the catalytic activity is required for some steps in the processing of recombination intermediates. However, overall, the formation of bivalents (connected parental chromosomes) was more successful in the catalytically inactive mutant than in the null mutant, as reflected in its significantly improved egg hatching rate. In contrast to the null mutant, we found that COs are enriched at chromosome arms in the *hd him*-6 mutant, similar to the wild type. Finally, our data indicate that the Bloom helicase supports the architecture of the late-recombination intermediates. We found that in *hd him-6* mutants, the distribution of MutSγ at late recombination sites is altered, suggesting that dHJ conformation is affected. Our epistasis experiments are consistent with a model in which these alterations impair the downstream processing of the joint molecule.

## Materials and methods

### Culture conditions

A complete list of *C. elegans* strains used in this study can be found in Supplementary Table S1. All strains were cultured at 20°C using standard methods [29].

### Immunofluorescence

Immunofluorescence was performed as previously described [30]. Briefly, L4-stage worms were picked the day before the experiment, incubated at 20°C for 20–24 h, and dissected in cutting buffer (1× phosphate buffered saline (PBS) containing 1.4 mM Levamisole) on a poly-l-lysine-coated SuperFrost slide. After dissection, fixing buffer (1% PFA (paraformaldehyde) in 1× PBS) was applied to the slide. The slide was incubated at room temperature for 5 min and then freeze-cracked in liquid nitrogen. The coverslip was removed and the slide was incubated in methanol at −20°C for 2 min, followed by two washing steps in 1× PBST [1× PBS containing 0.1% Tween] and blocking for 1 h in 1% bovine serum albumin (BSA) (dissolved in PBST) at room temperature. The primary antibody was added and the slide was incubated at 4°C overnight, washed three times in 1× PBST and incubated with the secondary antibody for 2 h at room temperature or for 1 h at 37°C for samples prepared for STED imaging. After washing with 1× PBST, the sample was incubated with 4′,6-diamidino-2-phenylindole (DAPI) (2 μg/ml) or with SYBR^™^ Gold Nucleic Acid Gel Stain (1:600 000) (Catalog number S11494) (in the case of samples prepared for STED imaging) for 1 min. Finally, the slide was washed once in 1× PBST for 20 min and mounted with Vectashield Mounting Medium (Vector Labs #H-1000). Samples prepared for STED imaging were mounted in Invitrogen ProLong^™^ Glass Antifade Mountant (Catalog number P36980) and incubated at 37°C for 1 h. A list of antibodies used can be found in Supplementary Table S2.

- Meiotic spreads

Samples were prepared as previously described [31]. Briefly, worms were dissected in 0.1% Tween-20, spread onto coverslips in a fixing solution (4% paraformaldehyde, 3.2% sucrose, 1% lipsol, and 1% sarcosyl), and dried overnight at room temperature. Coverslips were then incubated in methanol at −20°C for 20 min, washed three times in 1× PBST, and incubated with blocking solution (1% BSA in 1× PBST) for 30 min. The appropriate primary antibody was added and samples were incubated at 4°C overnight. After washing three times with 1× PBST, the procedure continued as described above.

- GFP::MSH-5 detection

Worms were dissected in cutting buffer (1× PBST) on poly-l-lysine-coated SuperFrost slides, immediately freeze-cracked in liquid nitrogen, incubated in methanol at −20°C for 20 min, and then incubated in fixation buffer (100 mM phosphate buffer containing 4% paraformaldehyde (PFA) at room temperature for 15 min. After three 5 min washes in 1× PBST, samples were counterstained with DAPI, washed, and mounted as described above.

### Imaging and quantification

Images were acquired using a DeltaVision Ultra microscope (×100 oil immersion objective lens; N.A. 1.4) equipped with a cMOS (complementary metal oxide semiconductor) camera. Stacks of 0.2 μm were taken and deconvolved with SoftWoRx software using the conservative mode coupled with 15 iterations. Maximum projections of the stacks were obtained after subtracting the background with a rolling ball of radius 50 pixels using ImageJ/Fiji [32]. The projected images were further adjusted for intensity levels using Adobe Photoshop, as previously described [33].

High-resolution images were acquired using a Zeiss Elyra 7 Structured Illumination Microscopy (SIM) microscope (×63 oil immersion objective lens) equipped with sCMOS cameras. Images were obtained with optimal spacing, channels aligned, correction matrices obtained from images acquired from TetraSpeck beads (T7279, ThermoFisher), SIM^2^ processed using ZenBlack software (weak live option), further projected with maximum intensity in Fiji, and adjusted for levels of intensity in Adobe Photoshop, as described above.

Stimulated emission depletion (STED) microscopy images were acquired using Abberior Instruments STEDYCON with an alpha Plan-Apochromat ×100/1.46 Oil DIC. Images acquired from STED/SIM were then segmented using the SYBR Gold channel/stained-SYP-1 channel, respectively, as a reference using the segmentation module available in Cell-ACDC [34]. SpotMAX [35] was utilized for detection of GFP::RMH-1, HIM-6::HA and OLLAS::COSA-1 foci. Only GFP::RMH-1/HIM-6::HA foci within a 150 nm radius of OLLAS::COSA-1 were counted. If the sigma value of the fitted Gaussian curve of a singlet of HIM-6::HA had a value ≥75.12 nm in either the *x*- or *y-*direction (as given by the SpotMAX parameters sigma_x_fit and sigma_y_fit respectively), it was classified as an elongated singlet. Parameters used for identification of these foci were kept constant for all images within an experiment.

### Viability assay

L4-stage worms were incubated at 20°C for 24 h and then transferred to fresh plates. The number of eggs laid and larvae hatched were counted 24 h after removal of the hermaphrodite parent. Unfertilized eggs were not counted. This transfer was repeated four times at 24-h intervals.

Viability was calculated as the number of larvae divided by the brood size (total number of larvae and the eggs counted). The number of male offspring was also counted.

### Recombination frequency assay

Bristol and Hawaiian strains of the mutants (*him-6(ok412)* and *him-6^K275R^*) and respective controls were crossed to create a hybrid, as previously described [21]. Hermaphrodites from the hybrid strain were crossed to males of a Bristol strain expressing a visible marker (EG7901). Five single nucleotide polymorphisms (SNPs) present on chromosome V (positions shown in Fig. 3C) were then tracked in progeny expressing the visible marker. Polymerase chain reaction (PCR) amplicons of the SNP-containing regions were digested with Dra1.

### Strain generation using CRISPR (Clustered regularly interspaced short palindromic repeats)

Strains were generated by CRISPR (Clustered regularly interspaced short palindromic repeats)–Cas9 using guide RNAs and repair templates (sequences shown below) according to a published protocol [36]. The strains generated by CRISPR were sequenced to verify that they contained the expected sequence alteration and outcrossed twice before being used in experiments.

Guide RNA and the repair template were obtained from Integrated DNA Technologies (IDT) and used to create the *him-6^K275R^*, *him-6^K275R^::ha*, *him-6^K275A^::ha*, and *him-6^K275R^* strains in the Hawaiian background.

Reagents used to make the *him-6^K275R^, him-6^K275R^::ha* and *him-6^K275R^* mutants were:

- Guide RNA (Alt-R^®^ CRISPR–Cas9 CRISPR ribonucleic acid (crRNA), 10 nmol)–5′-GTT CTT ATG CCA ACT GGC GC-3′
- Repair template (Ultramer^®^ DNA Oligo, 4 nmol)–5′-GTA TCC TTT CTA CAT TAA TGG GCC ACG ACA CCT TTG TGC TCA TGC CTA CTG GGG CGG GAA GAA GTT TGT GCT ACC AAT TGC CAG CTG TCA TTC TTC C-3′
- Reagents used to make the *him-6^K275A^::ha* mutant were:
- guide RNA (Alt-R^®^ CRISPR–Cas9 crRNA, 10 nmol)–as shown above
- Repair template (Ultramer^®^ DNA Oligo, 4 nmol)–5′-GTA TCC TTT CTA CAT TAA TGG GCC ACG ACA CCT TTG TGC TCA TGC CTA CTG GGG CGG GAG CGA GTT TGT GCT ACC AAT TGC CAG CTG TCA TTC TTC C-3′

### Western blotting

L4-stage worms (200) were pre-picked and incubated at 20°C, collected in M9 buffer after 20–24 h, and washed three times. Sample buffer was added to the worm pellet, and boiled for 10 min at 99°C, followed by snap-freezing in liquid nitrogen. The boiling/freezing cycle was repeated three times. Samples were loaded onto a precast 4%–20% gradient polyacrylamide gel (Bio-Rad Laboratories, cat. #4561094) under denaturing conditions [1× sodium dodecyl sulfate (SDS) running buffer: 0.2501 M Tris, 1.924 M glycine, and 0.0347 M SDS]. Resolved proteins were transferred to a polyvinylidene fluoride (PVDF) membrane for 1 h at 4°C and 100 V in 1× Tris-glycine buffer (125 mM Tris and 960 mM glycine) containing 20% methanol. After washing three times with 1× TBST (0.1% Tween-20), the membrane was blocked in 5% skimmed milk for 30 min and then incubated overnight on a platform shaker at 4°C with the appropriate primary antibody. The membrane was washed three times in 1× TBST and incubated for 2 h with the horseradish peroxidase (HRP)-conjugated secondary antibody at room temperature on a platform shaker. The membrane was then exposed to the ECL Star Enhanced Chemiluminescent Substrate (Euroclone cat #EMP001005) for 1 min (to visualize low-abundance proteins) or for 1 min with WesternBright Sirius HRP substrate (Advansta K-12043-D20) and imaged with a ChemiDoc system (Bio-Rad Laboratories). ChemiDoc images were analyzed using Image Lab software and intensity calculation were done in Fiji [32].

### Quantification of apoptotic nuclei with SYTO12 staining

L4-stage worms were incubated at 20°C for 20–24 h to obtain young adults. They were then incubated at 20°C for 2 h in 33 mM SYTO12 solution, transferred to seeded Nematode Growth Medium (NGM) plates, and allowed to recover for 20 min before being placed on 2% agarose-padded slides with 40 mM Levamisole in M9 buffer. Apoptotic nuclei were counted using a Zeiss Axio microscope equipped with a ×40 or ×63 oil immersion objective lens.

### Single-molecule localization microscopy

Samples were prepared for single-molecule localization microscopy (SMLM) as previously described [37] using a 1:200 dilution of primary antibody (Ck-anti-HTP-3 [38]; and Ms-a-V5, Thermo Fisher Scientific, R960-25, mouse monoclonal) and a 1:50 dilution of secondary F(ab’)_2_-anti-mouse antibody (Jackson ImmunoResearch, AB_2 340 761, donkey polyclonal) conjugated to Alexa Fluor 647 (Thermo Fischer Scientific, A37573) or F(ab’)_2_-anti-chicken antibody (Jackson ImmunoResearch, AB_2 340 347, donkey polyclonal) conjugated to CF660C (Biotium, 92137).

Images were acquired at room temperature using a custom-built 3-dimensional (3D)-SMLM microscope [39] with a UPLSAPO100XS [numerical aperture (NA) 1.35] silicon oil objective and a cylindrical lens for 3D imaging. Ratiometric dual-color acquisition [40, 41] was achieved by splitting the emission light with a 665 nm long-pass dichroic mirror and filtering transmitted and reflected photons with 700/100 nm and 676/37 nm filters, respectively.

SMLM images were reconstructed and post-processed using SMAP software (Super-resolution Microscopy Analysis Platform) [42] following standard procedures [43, 44]. The globLoc algorithm [45] was used to reconstruct SMLM image from the raw images based on an experimental point spread function model and by linking emitter coordinates across the two channels. The point spread function model and channel transformation were generated from *z*-stack images of fluorescent beads (TetraSpeck Microspheres, T7279, Thermo Fisher Scientific) using the calibrate3DsplinePSF plugin.

Sample drift was corrected using the redundant cross-correlation algorithm within SMAP [42, 43]. Dim and out-of-focus emitters were excluded from further analysis. They were identified as having *z*-coordinates outside a 600 nm window or a localization precision of >15 nm in *xy* plane or >25 nm along the *z* axis. Super-resolved images were rendered in SMAP, with each localization represented by a Gaussian function with a width corresponding to its precision [42].

To describe the size and shape of MSH-5::V5 foci, we selected regions containing foci along chromosome axes oriented in the frontal or a slightly tilted view. MSH-5::V5 localizations were fitted to a hollow elliptical ring model from the LocMoFit framework in SMAP [46]. The fit was initialized with an elliptical model whose center was placed at the median coordinates of MSH-5::V5 localizations and whose major and minor axes were 65 and 35 nm, respectively. To compare the shapes, we evaluated the fit results from the hollow elliptical model and compared them to fitting a custom model of a filled ellipse. To determine which of the two models fits best, we use the corrected and normalized Akaike Information Criterion (normAICc), as given by LocMoFit, where lower values indicate greater confidence in fitting the data to the given model. The foci are designated “filled” when the normAICc_filled is smaller than normAICc_hollow.

### Auxin-induced depletion of TOP-3

L4-stage worms from the appropriate strains were picked and incubated at 20°C to obtain young adults. The young adults were placed on agar plates containing either 1 mM indole-3-acetic acid (auxin) or ethanol (solvent control) and seeded with a suspension of OP50 *Escherichia coli* and 1 mM of auxin in ethanol. Worms were incubated for 40 h on the plates before being dissected and processed as described for “Immunofluorescence”.

## Results and discussion

### Meiosis is more successful in catalytically inactive *him-6* mutants than in null mutants

The BTR complex contributes to both CO-promoting and CO-antagonizing activities at multiple steps of meiotic recombination [16]. *rmh-1* (RMI1 ortholog), *rmif-2* (RMI2 ortholog), and *him-6* (Bloom helicase ortholog) mutants display remarkable numbers of univalents in diakinesis—in contrast to *top-3* mutants, as shown here (Supplementary Fig. S1A) and reported previously [5, 16, 21, 47]. The presence of univalents in diakinesis indicates aberrant or lack of CO formation. In contrast, wild type worms exhibit robust CO formation resulting in connected parental homologous chromosomes that can be distinguished as six bivalents in DAPI-stained diakinesis oocytes. To find out whether HIM-6 exerts a structural or a catalytic role during CO formation, we generated catalytically inactive *him-6* mutants. In *Saccharomyces cerevisiae*, a helicase inactive mutant of Sgs1 was created by mutating the invariant lysine in the Walker-A motif within the Sgs1 ATPase domain [48]. Similarly, in *C. elegans* HIM-6, *in vitro* studies have shown that mutating the corresponding conserved lysine in the ATPase domain to alanine renders it incapable of hydrolyzing ATP and, therefore, unable to unwind forked duplex substrates [49].

**Figure 1.**
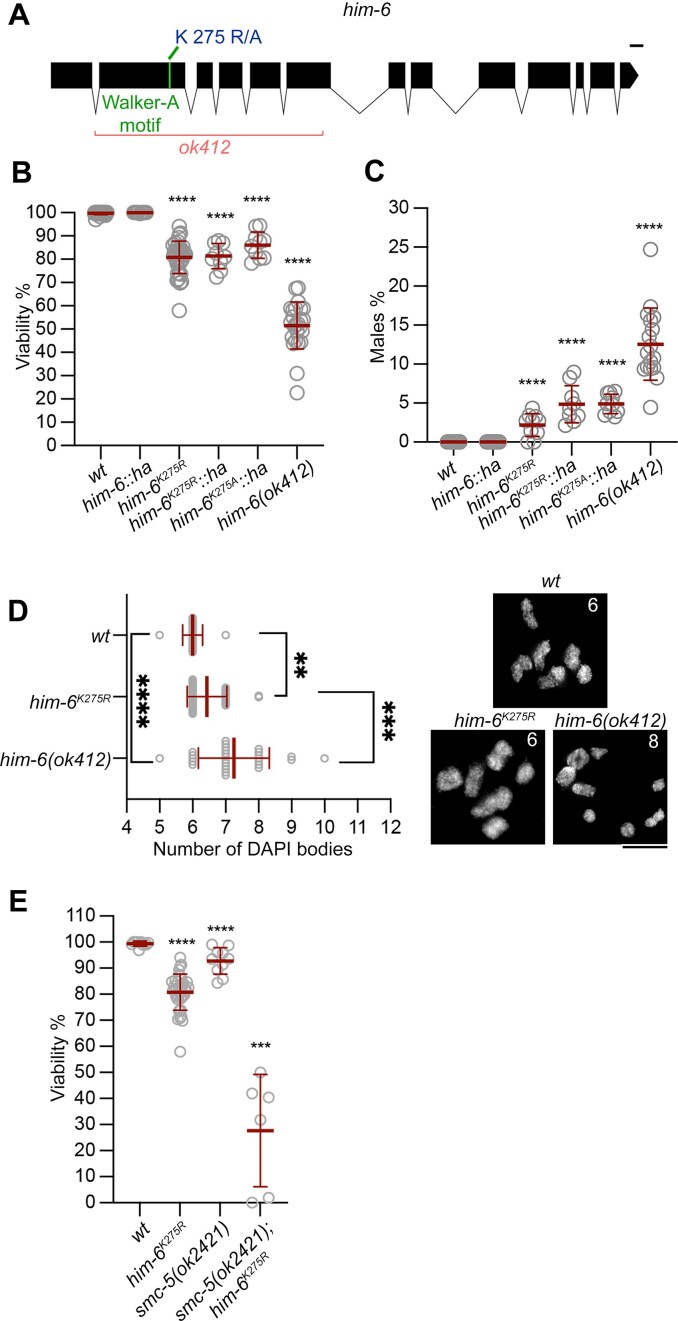
Meiosis is less compromised in catalytically inactive than in deletion *him-6* mutants. (**A**) Schematic representation of the *him-6* gene locus, with shaded boxes indicating exons and lines indicating intronic sequences. The deleted region in the *him-6(ok412)* allele is shown. The Walker-A motif containing the invariant lysine-275 that was mutated to either alanine or arginine is labeled in green. The scale bar represents 100 bases. (**B**) Viability counts in various genotypes. The number of worms used (*n*) and percentage viability (mean ± SD) for each genotype were: wild type *(wt)*, *n* = 37, 99.65 ± 0.59%; *him-6::ha*, *n* = 9, 99.95 ± 0.15%; *him-6^K275R^*, *n* = 38, 80.77 ± 6.95%; *him-6^K275R^::ha*, *n* = 9, 81.33 ± 5.48%; *him-6^K275A^::ha*, *n* = 10, 86.06 ± 5.63%; and *him-6(ok412)*, *n* = 25, 51.51 ± 10.04%. The Mann–Whitney test was used to identify statistically significant differences between genotypes: ****P< 0.0001, ***P< 0.001, **P< 0.01, *P< 0.05, and not significant (ns), *P*≥ 0.05. Unless otherwise indicated, all comparisons were with the wild type. The statistical significance (*P-*value) of differences between pairs of genotypes was: **** (<0.0001) for all pairs except *him-6^K275R^* versus *him-6^K275A^::ha*, * (0.0451); *wt* versus *him-6::ha*, ns (0.0633); *him-6^K275R^* versus *him-6^K27AR^::ha*, ns (0.9049); and *him-6^K275R^::ha* versus *him-6^K275A^::ha*, ns (0.0947). (**C**) Comparison of the percentage of males among the offspring for various genotypes. The number of worms used (*n*) and percentage of males (mean ± SD) for each genotype were: wild type (*wt)*, *n* = 19, 0.00 ± 0.00%; *him-6::ha*, *n* = 9, 0.00 ± 0.00%; *him-6^K275R^*, *n* = 10, 2.19 ± 1.46%; *him-6^K275R^::ha*,*n* = 9, 4.86 ± 2.39%; *him-6^K275A^::ha*, *n* = 10, 4.90 ± 1.25%; and *him-6(ok412)*, *n* = 17, 12.56 ± 4.63%. The Mann–Whitney test was used to compare percentage of males between genotypes: ****P< 0.0001, ***P< 0.001, **P< 0.01, *P< 0.05, and not significant (ns), *P*≥ 0.05. The statistical significance (*P-*value) of differences was: *wt* versus *him-6::ha*, ns (>0.9999); wt versus *him-6^K275R^*, **** (<0.0001); *wt* versus *him-6^K275R^::ha*, **** (<0.0001); *wt* versus *him-6^K275A^::ha*, **** (<0.0001); *wt* versus *him-6(ok412)*, **** (<0.0001); *him-6::ha* versus *him-6^K275R^*, *** (0.0007); *him-6::ha* versus *him-6^K275R^::ha*, **** (<0.0001); *him-6::ha* versus *him-6^K27AR^::ha*, **** (<0.0001); *him-6::ha* versus *him-6(ok412)*, **** (<0.0001); *him-6^K275R^::ha* versus *him-6(ok412)*, **** (<0.0001); *him-6^K275R^* versus *him-6^K27AR^::ha*, * (0.0168); *him-6^K275R^* versus *him-6^K275A^::ha*, *** (0.0003); *him-6^K275R^*versus*him-6(ok412)*, **** (<0.0001); *him-6^K275R^::ha* versus *him-6(ok412)*, **** (<0.0001); *him-6^K275A^::ha* versus *him-6(ok412)*, **** (<0.0001); and *him-6^K275R^::ha* versus *him-6^K275A^::ha*, ns (0.6175). (**D**) Left panel, graph showing the number of DAPI-bodies in diakinesis oocytes. The number of oocytes counted (*n*) and number of DAPI bodies (mean ± SD) were: wild type (*wt)*, *n* = 23, 6.00 ± 0.30; *him-6^K275R^*, *n* = 39, 6.44 ± 0.60; and *him-6(ok412)*, *n* = 28, 7.25 ± 1.08. The Mann–Whitney test was used to assess the statistical significance (*P*-value) of differences between genotypes: *wt* versus *him-6^K275R^*, ** (0.0024); *wt* versus *him-6(ok412)*, **** (<0.0001); and *him-6(ok412)* versus *him-6^K275R^*, *** (0.0002). Right panel, representative examples of DAPI bodies in the -1 oocyte in diakinesis, with full projection of the images. The number of DAPI bodies counted in each example is indicated. The scale bar represents 5 μm. (**E**) Graph showing the percentage viability of the indicated genotypes. The number of worms used (*n*) and percentage viability (mean ± SD) were: wild type (*wt*), *n* = 10, 99.33 ± 0.97%; *smc-5(ok2421)*, *n* = 10, 92.76 ± 5.08%; *him-6^K275R^*, *n* = 38, 80.77 ± 6.95%; and *smc-5(ok2421) him-6^K275R^*, *n* = 6, 27.68 ± 21.51%. The Mann–Whitney test was used to compare mutant genotypes with the *wt*: ****P< 0.0001, ***P< 0.001, **P< 0.01, *P< 0.05, and not significant (ns), *P*≥ 0.05. The statistical significance (*P*-value) of differences between genotypes was: *wt* versus *smc-5(ok2421)*, **** (<0.0001); *wt* versus *him-6^K275R^*, **** (<0.0001); *wt* versus *smc-5(ok2421); him-6^K275R^*, *** (0.0002); *him-6^K275R^* versus *smc-5(ok2421)*, **** (<0.0001); and *smc-5(ok2421); him-6^K275R^* versus *smc-5(ok2421)*, *** (0.0002).

**Figure 2.**
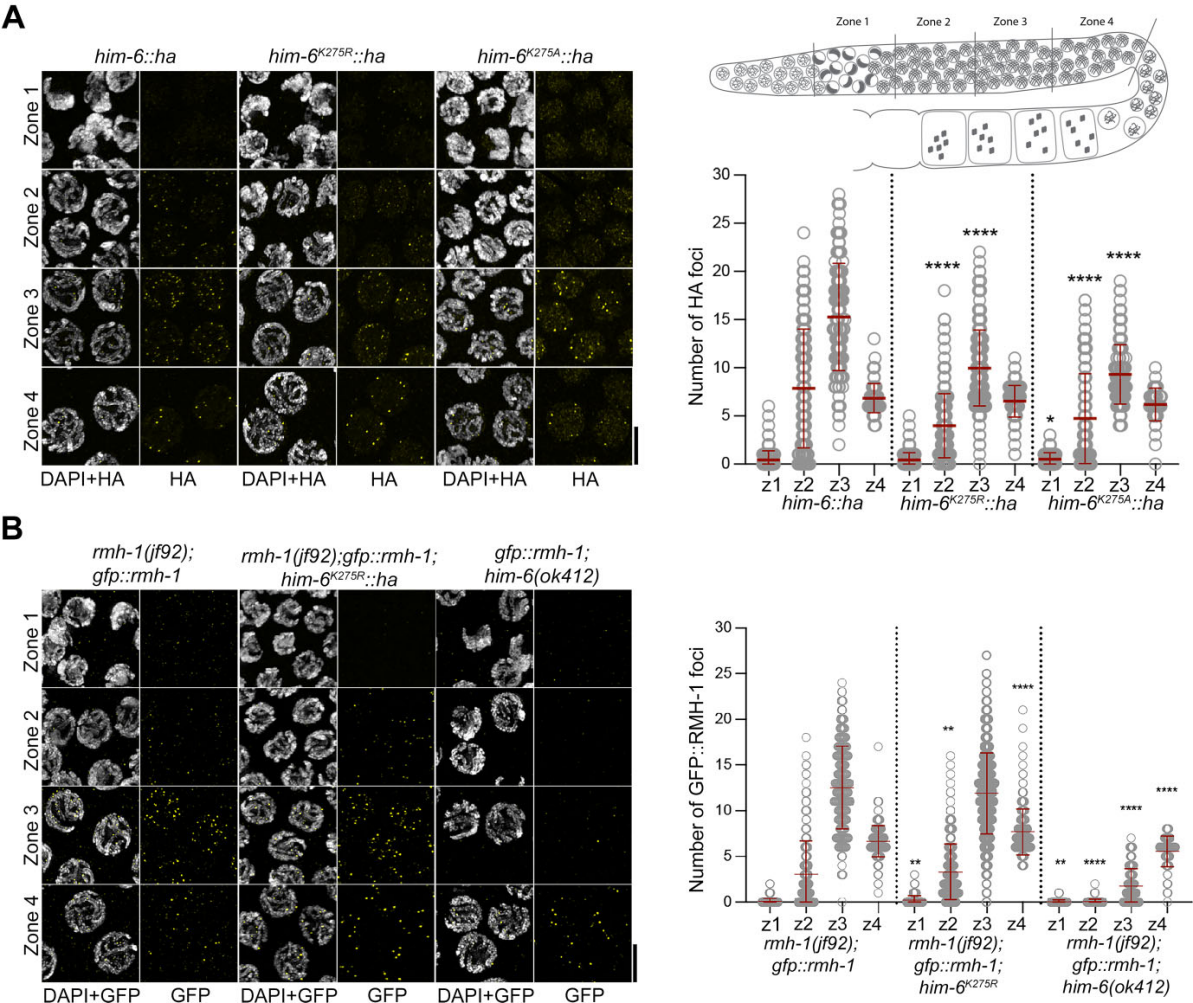
In *him-6^K275R^::ha*, HIM-6 foci dynamics resembles the wild type and early RMH-1 localization is independent of HIM-6 helicase activity. (**A**) Left panel, representative examples of nuclei in zones 1–4 of the indicated genotypes, labeled with anti-HA antibody (yellow) and counterstained with DAPI (gray). The scale bar represents 5 μm. Brightness levels on the pictures were adjusted for better visualization of the foci. Upper right panel, schematic representation of the four zones defined on the germline, from meiotic entry to late pachynema. Lower right panel, a graph showing the number of HA foci counted in the indicated strains each zone (mean ± SD). The number of nuclei counted in each zone (*n*) and number of foci (mean ± SD) were as follows. *him-6::ha*: zone 1, *n* = 141, 0.44 ± 0.96; zone 2, *n* = 171, 7.85 ± 6.17; zone 3, *n* = 132, 15.27 ± 5.56; and zone 4, *n* = 91, 6.85 ± 1.51. *him-6^K275R^::ha*: zone 1, *n* = 282, 0.44 ± 0.74; zone 2, *n* = 279, 3.98 ± 3.32; zone 3, *n* = 266, 9.97 ± 3.94; and zone 4, *n* = 137, 6.53 ± 1.64. *him-6^K275A^::ha*: zone 1, *n* = 142, 0.50 ± 0.68; zone 2, *n* = 150, 4.73 ± 4.66; zone 3, *n* = 121, 9.31 ± 3.09; and zone 4, *n* = 53, 6.2 ± 1.70. The Mann–Whitney test was used to compare foci numbers in mutants with the wild type (*him-6::ha*): ****P< 0.0001, ***P< 0.001, **P< 0.01, *P< 0.05, and not significant (ns), *P*≥ 0.05. The statistical significance (*P*-value) of differences in HA foci number for each zone between each mutant and the *wt* is given in the following order: *him-6::ha* versus *him-6^K275R^::ha*, *him-6::ha* versus *him-6(ok412)*, and *him-6^K275R^::ha* versus *him-6(ok412)*. Zone 1: ns (0.1754), * (*P*= 0.0206), and ns (0.1808). Zone 2: **** (<0.0001), **** (<0.0001), and ns (0.9426). Zone 3: **** (<0.0001), **** (<0.0001), and ns (0.1438). Zone 4: ns (0.5112), ns (0.0753), and ns (0.2141). (**B**) Left panel, representative examples of nuclei in zones 1–4 of the indicated genotypes. The scale bar represents 5 μm. Right panel, graph showing the number of GFP::RMH-1 foci counted in each zone (mean ± SD). The number of nuclei counted in each zone (*n*) and number of foci (mean ± SD) were as follows: *rmh-1(jf92)*;*gfp::rmh-1*: zone 1, *n* = 243, 0.09 ± 0.32; zone 2, *n* = 301, 3.03 ± 3.65; zone 3, *n* = 279, 12.55 ± 4.5; and zone 4, *n* = 205, 6.65 ± 1.72. *rmh-1(jf92); gfp::rmh-1; him-6^K275R^::ha*: zone 1, *n* = 246, 0.21 ± 0.5; zone 2, *n* = 309, 3.31 ± 3.04; zone 3, *n* = 330, 11.9 ± 4.45; and zone 4, *n* = 215, 7.69 ± 2.5. *gfp::rmh-1; him-6(ok412)*: zone 1, *n* = 98, 0.04 ± 0.2; zone 2, *n* = 156, 0.05 ± 0.25; zone 3, 1.8 ± 1.9; zone 4, *n* = 85, 5.55 ± 1.68. The Mann–Whitney test was used to compare foci numbers in mutants with the wild type (*rmh-1(jf92); gfp::rmh-1*): ****P< 0.0001, ***P< 0.001, **P< 0.01, *P< 0.05 and not significant (ns), *P*≥ 0.05. The statistical significance (*P-*value) of differences in GFP::RMH-1 foci number in each zone between mutant and *wt* strains is given in the following order: *wt* versus *rmh-1(jf92); gfp::rmh-1*; *him-6^K275R^::ha*, *wt* versus *gfp::rmh-1*; *him-6(ok412)*; and *rmh-1(jf92); gfp::rmh-1; him-6^K275R^::ha* versus *gfp::rmh-1; him-6(ok412)*. Zone 1: ** (0.0011), ns (0.2823), and ** (0.0011). Zone 2: ** (0.0032), **** (<0.0001), and **** (<0.0001). Zone 3: ns (0.0823), **** (<0.0001), and **** (<0.0001). Zone 4: **** (<0.0001), **** (<0.0001), and **** (<0.0001).

CRISPR–Cas9 [36] was used to mutate lysine-275 of *him-6* to arginine in wild type and *him-6::ha* worms (resulting in *him-6(jf209[him-6(K275R)])*and*him-6(jf275[him-6(K275R)::ha])*) and to alanine in *him-6::ha* worms (resulting in *him-6[jf269(him-6(K275A)::ha]* (Fig. 1A). Comparable to budding yeast Sgs1 [15], the two mutant strains were not significantly different from each other regarding embryo viability, percentage of males among the offspring, and number of HA-tagged HIM-6 foci (Figs 1B, C, and 2A). Therefore, all further experiments used *him-6(jf209)* and *him-6(jf275)::ha*, hereafter referred to as *him-6^K275R^* and *him-6^K275R^::ha*, respectively.

Embryo viability was remarkably higher in the *him-6^K275R^* mutant (80.77%) than in the null mutant *him-6(ok412)* (51.51%) but lower than in the wild type (99.65%, Fig. 1B). A higher percentage of males indicates X chromosome segregation defects [50]. Phenotypic analysis revealed that, the percentage of males in the *him-6^K275R^* mutant was lower than in the null mutant but slightly higher than in the wild type (mean ± SD of the percentage of males: wild type (*wt*), 0.00 ± 0.00%; *him-6^K275R^*, 2.19 ± 1.46%; and *him-6(ok412)*, 12.56 ± 4.63%) (Fig. 1C).

We also quantified the number of DAPI bodies in the -1 oocyte in diakinesis as a proxy for the fidelity of upstream meiotic processes, including CO formation. DAPI body counts in *him-6^K275R^* were more similar to wild type counts (mean ± SD of DAPI bodies: *wt*, 6.00 ± 0.30; *him-6^K275R^*, 6.44 ± 0.60) than to the elevated counts in the null mutant *him-6(ok412)* (7.25 ± 1.08; Fig. 1D).

*him-6(ok412)* is synthetic lethal with *smc-5* mutants, and a model proposes that the aberrant DNA intermediates formed in the absence of SMC-5 are eliminated by HIM-6 unwinding activity [5]. Therefore, we generated the double mutant *smc-5(ok2421); him-6^K275R^*. In the double mutant, embryo viability was severely reduced to 27.7 ± 21.5%, which is significantly lower than in the wild type or any of the single mutants (Fig. 1E), providing strong evidence that *him-6^K275R^* mutants encode a catalytically inactive HIM-6 protein.

In summary, the *him-6^K275R^* mutant displays an intermediate phenotype between the null allele and wild type regarding the fidelity of chromosome segregation, egg hatching rates, and bivalent formation. The strong synthetic lethality with *smc-5* mutants provides further evidence that the *him-6^K275R^ hd* mutant is defective in unwinding aberrant recombination intermediates.

### RMH-1 (*C. elegans* RMI1) localization to recombination intermediates is independent of HIM-6 helicase activity

To determine whether helicase-dead HIM-6 protein is capable of localizing to recombination intermediates, we immunostained *him-6::ha* and *him-6^K275R^::ha* with anti-HA. HIM-6 localization dynamics in both *him-6^K275R^::ha* and *him-6^K275A^::ha* recapitulated the reported pattern of a peak in mid-pachynema, followed by a reduction in foci numbers in late pachynema in *him-6::ha* (Fig. 2A) [16]. For foci quantification, the gonad was divided into four equal zones from the transition zone until late pachynema (schematic shown in Fig. 2A), with zone 1 corresponding to the transition zone, zone 2 to early pachynema, zone 3 to mid-pachynema, and zone 4 to late pachynema. The mean (± SD) number of HIM-6 foci in zone 2 [wild type (*wt*), 7.85 ± 6.17; *him-6^K275R^::ha*, 3.98 ± 3.32; and *him-6^K275A^:: ha*, 4.73 ± 4.66] and zone 3 (*wt*, 15.27 ± 5.56; *him-6^K275R^::ha*, 9.97 ± 3.94; and *him-6^K275A^:: ha*, 9.31 ± 3.09) indicated a significant reduction of foci in the mutants. However, in zone 4, the number of foci was similar for all three genotypes (*wt*, 6.85 ± 1.51; *him-6^K275R^::ha*, 6.53 ± 1.64; and *him-6^K275A^:: ha*, 6.2 ± 1.7). Overall, the foci were dimmer in the *hd* mutant (Supplementary Fig. S2A). Western blot analysis of lysed adult worms suggested that the wild type and *hd* mutant contain similar amounts of HIM-6 protein (Supplementary Fig. S2B), suggesting that the mutation does not affect HIM-6 protein stability.

Like other components of the BTR complex (HIM-6, TOP-3, and RMIF-2), RMH-1 begins to appear as foci in early pachynema, with foci numbers peaking in mid-pachynema and reducing to around six in late pachynema [16, 21, 31, 47]. The distinct RMH-1 foci formation in early and mid-pachynema was previously shown to depend on the presence of HIM-6, whereas late pachynema foci occur independently of *him-6* activity [16]. Therefore, we investigated whether the absence of helicase activity in HIM-6 would affect RMH-1 localization in early and mid-pachynema. Consistent with previous reports, GFP::RMH-1 foci numbers were significantly reduced in early and mid-pachynema in the null mutant *him-6(ok412)* (zone 2, 0.05 ± 0.25; zone 3, 1.8 ± 1.9; Fig. 2B). Surprisingly, early GFP::RMH-1 foci numbers were not reduced by as much in the *him-6^K275R^::ha* mutant (zone 2, 3.31 ± 3.04; zone 3, 11.9 ± 4.45; Fig. 2B), demonstrating that the early localization of RMH-1 is independent of HIM-6 helicase activity.

In summary, the signal intensity and number of HIM-6 foci were reduced in helicase-dead mutants. These results show that HIM-6 helicase activity is largely dispensable for RMH-1 localization to recombination foci, unlike in *him-6(ok412)* (Fig. 2B) [16], where the reduction is more pronounced. Nevertheless, HIM-6 helicase activity is required to achieve wild type numbers of HIM-6 and RMH-1 foci.

### Dynamics of recombination marker expression and apoptosis levels in the *him-6* catalytically inactive mutant is similar to in the wild type

Given the significant improvement in hatching rates observed in the him-6^K275R^ mutant, we next investigated whether the recombination rate is higher in this mutant than in the null mutant *him-6(ok412)*. SPO-11-induced DNA DSBs are resected and then coated with the RAD-51 recombinase to facilitate inter-homolog invasions [2]. These recombination intermediates are later stabilized by MSH-5, and some mature into COs in a *msh-5*- and *cosa-1*-dependent manner [51, 52]. The appearance of MSH-5 and COSA-1 foci on recombination intermediates starts in early pachynema, with foci numbers peaking in mid-pachynema and reducing to an average of six foci in late pachynema [51–53]. We first compared the dynamics of RAD-51 foci in *him-6^K275R^* and *him-6(ok412)* with the wild type by quantifying the foci in gonads divided into seven zones from the distal tip to late pachynema (Supplementary Fig. S3A). In *him-6(ok412)*, RAD-51 foci persisted until zone 7 (slightly longer than in the wild type), corresponding to late pachynema, as previously reported [28]. In contrast, RAD-51 foci in *him-6^K275R^* mutants did not persist until zone 7 (Supplementary Fig. S3A and B, and Supplementary Table S3).

**Figure 3.**
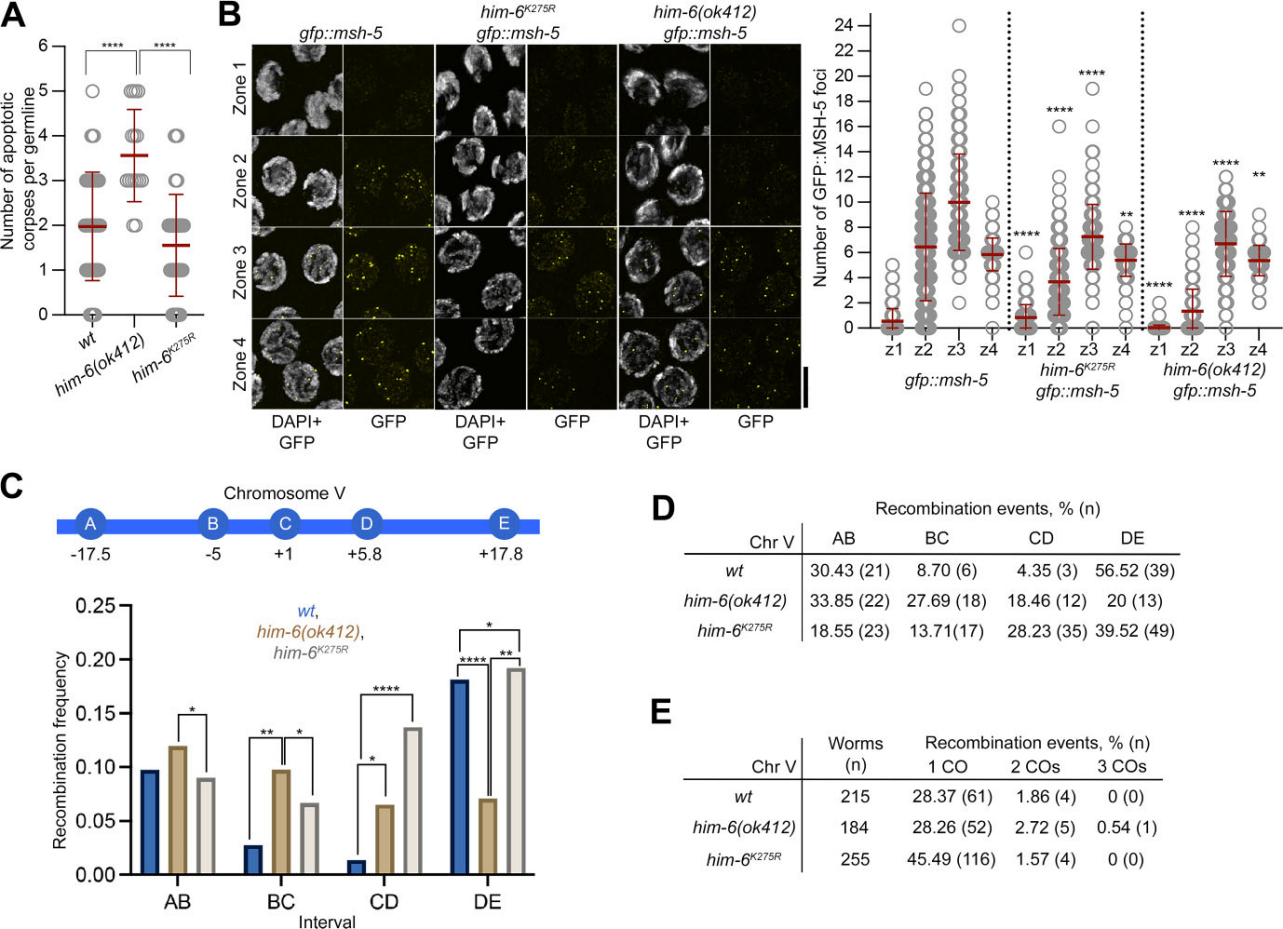
The apoptosis levels in *him-6^K275R^* mutants are similar to wild type and recombination frequency is elevated in *him-6^K275R^*mutants. (**A**) SYTO-12-positive germline nuclei (indicating apoptotic corpses) were counted in the indicated genotypes. The number of gonads assessed (*n*) and number of SYTO-12-positive nuclei per gonad (mean ± SD) were: wild type(*wt*), *n* = 44, 1.98 ± 1.21; *him-6(ok412)*, *n* = 16, 3.56 ± 1.03; and *him-6^K275R^*, *n* = 47, 1.55 ± 1.14. The Mann–Whitney test was used to compare numbers of SYTO-12-positive nuclei per gonad in the mutant and wild type: ****P< 0.0001, ***P< 0.001, **P< 0.01, *P< 0.05, and not significant (ns), *P*≥ 0.05. The statistical significance (*P*-value) of differences between genotypes was: *wt* versus *him-6^K275R^*, ns (0.0811); *wt* versus *him-6(ok412)*, **** (<0.0001); and *him-6^K275R^* versus *him-6(ok412)*, **** (<0.0001). (**B**) Left panel, representative images of DAPI and GFP::MSH-5 signals in germline nuclei of the *gfp::msh-5, him-6^K275R^ gfp::msh-5*, and *him-6(ok412) gfp::msh-5* genotypes from zone 1 (meiotic entry) to zone 4 (late pachynema). Zones are defined in Fig. 2A. Right panel, graph showing the number of GFP::MSH-5 foci in the indicated genotypes. The scale bar represents 5 μm. The number of nuclei assessed in each zone (*n*) and number of GFP::MSH-5 foci (mean ± SD) were as follows. *gfp::msh-5*: zone 1, *n* = 324, 0.55 ± 1.01; zone 2, *n* = 341, 6.45 ± 4.27; zone 3, *n* = 283, 10 ± 3.82; and zone 4, *n* = 171, 5.85 ± 1.31. *him-6^K275R^ gfp::msh-5*: zone 1, *n* = 309, 0.85 ± 1.01; zone 2, *n* = 291, 3.68 ± 2.65; zone 3, *n* = 237, 7.25 ± 2.57; and zone 4, *n* = 137, 5.39 ± 1.27. *him-6(ok412) gfp::msh-5*, zone 1, *n* = 167, 0.03 ± 0.2; zone 2, *n* = 186, 1.34 ± 1.76; zone 3, *n* = 144, 6.69 ± 2.57; and zone 4, *n* = 86, 5.36 ± 1.21. The Mann–Whitney test was used to compare foci numbers in zones 1–4 of mutant and wild type strains: ****P< 0.0001, ***P< 0.001, **P< 0.01, *P< 0.05, and not significant (ns), *P*≥ 0.05. The statistical significance (*P-*value) of differences between genotypes are given in the following order: *gfp::msh-5* versus *him-6^K275R^ gfp::msh-5*, *gfp::msh-5* versus *him-6(ok412) gfp::msh-5*, and *him-6^K275R^ gfp::msh-5* versus *him-6(ok412) gfp::msh-5*. Zone1: **** (<0.0001), **** (<0.0001), and **** (<0.0001). Zone 2: **** (<0.0001), **** (<0.0001), and **** (<0.0001). Zone 3: **** (<0.0001), **** (<0.0001), and ns (0.2893). Zone 4: ** (0.0033), ** (0.001), and ns (0.573). (**C**) Upper panel, schematic representation of the SNP positions on chromosome V used in the assay. Lower panel, graph showing the recombination frequency at intervals AB, BC, CD, and DE on chromosome V in the indicated genotypes. Fisher’s exact test was used to compare differences in recombination frequency for each interval between wild type (*wt*) and mutant genotypes: ****P< 0.0001, ***P< 0.001, **P< 0.01, *P< 0.05, and not significant (ns), *P*≥ 0.05. The statistical significance (*P*-value) of differences in recombination frequency were as follows. Interval AB: *wt* versus *him-6(ok412)*, ns (0.7138); *wt* versus *him-6^K275R^*, ns (0.0735); and *him-6(ok412)* versus *him-6^K275R^*, * (0.0301). Interval BC: *wt* versus *him-6(ok412)*, ** (0.0061); *wt* versus *him-6^K275R^*, ns (0.3602); and *him-6(ok412)* versus *him-6^K275R^*, * (0.0290). Interval CD: *wt* versus *him-6(ok412)*, * (0.0126); *wt* versus *him-6^K275R^*, **** (<0.0001); and *him-6(ok412)* versus *him-6^K275R^*, ns (0.1593). Interval DE: *wt* versus *him-6(ok412)*, **** (<0.0001); *wt* versus *him-6^K275R^*, * (0.0248); and *him-6(ok412)* versus *him-6^K275R^*, ** (0.0087). The total number of worms analyzed were: *wt*, 215; *him-6(ok412)*, 184; and *him-6^K275R^*, 255. (**D**) Table showing the number of recombination events for each interval on chromosome V (Chr V) and the percentage of the total in the indicated the genotypes. (**E**) Table showing the number of worms used and the number (and percentage) of one, two, and three CO events on chromosome V (Chr V) in the indicated genotypes.

Persistent DNA damage in the germline induces apoptosis [54]. Therefore, we compared the number of apoptotic corpses in the *him-6(ok412)* and *him-6^K275R^* mutants by SYTO-12 staining [55]. The number of apoptotic corpses was similar to the wild type in *him-6^K275R^* mutants, but was higher in the *him-6(ok412)* null mutant [mean ± SD: *wt*, 1.98 ± 1.21; *him-6(ok412)*, 3.56 ± 1.03; and *him-6^K275R^*, 1.55 ± 1.14, Fig. 3A]. This finding suggests that fewer aberrant intermediates are generated or that recombination proceeds further in the *him-6^K275R^* mutant than in *him-6(ok412)* (Fig. 3A and Supplementary Fig. S3A and B, and Supplementary Table S3).

We next compared whether the dynamics of the CO factors MSH-5 and COSA-1 are similar in the mutants and the wild type. For this, we cytologically examined the number of GFP::MSH-5 and OLLAS::COSA-1 foci in the tagged mutant strains (Fig. 3B, and Supplementary Fig. S3C and D). We divided the gonad into four equal zones from meiotic entry to late pachynema, with zones 2, 3, and 4 representing early, mid, and late pachynema, respectively. In all four zones, the numbers of GFP::MSH-5 foci in the mutants *him-6^K275R^ gfp::msh-5* and *him-6(ok412) gfp::msh-5* were significantly lower than in the wild type (Fig. 3B), especially in zone 2 (mean ± SD; *gfp::msh-5*, 6.45 ± 4.27; *him-6^K275R^ gfp::msh-5*, 3.68 ± 2.65; and *him-6(ok412) gfp::msh-5*, 1.34 ± 1.76), zone 3 (wild type *gfp::msh-5*, 10 ± 3.82; *him-6^K275R^ gfp::msh-5*, 7.25 ± 2.57; and *him-6(ok412) gfp::msh-5*, 6.69 ± 2.57), and zone 4 (wild type *gfp::msh-5*, 5.85 ± 1.31; *him-6^K275R^ gfp::msh-5*, 5.39 ± 1.27; and *him-6(ok412) gfp::msh-5*, 5.36 ± 1.21).

The pattern of OLLAS::COSA-1 foci was similar to the pattern observed with GFP::MSH-5 (Supplementary Fig. S3C and D). Most importantly, the number of OLLAS::COSA-1 foci in zone 4 was reduced for both mutants compared with the wild type (mean ± SD; *ollas::cosa-1*; *him-6::ha*, 6.32 ± 2.65; *ollas::cosa-1; him-6^K275R^::ha*, 5.55 ± 1.55; and *ollas::cosa-1; him-6(ok412*), 5.38 ± 1.58). The number of foci in zone 3 (corresponding to mid-pachynema) in *ollas::cosa-1; him-6^K275R^::ha* was slightly lower than in the wild type and further reduced in *ollas::cosa-1; him-6(ok412*) (mean ± SD; *ollas::cosa-1; him-6::ha*, 2.77 ± 2.89; *ollas::cosa-1; him-6^K275R^::ha*, 2.35 ± 2.21; and *ollas::cosa-1;him-6(ok412*), 1.58 ± 2.38).

The presence of CO-committed intermediates is known to stabilize the synaptonemal complex in *cis* and trigger asynapsis of chromosomes lacking such intermediates [56, 57]. This encouraged us to investigate the level of asynapsis in the mutants. We found that significantly fewer synapsed nuclei could be found in late pachynema in *him-6(ok412)* when compared to the wild type and *him-6^K275R^* [percentage of synapsed nuclei in zone 7, mean ± SD: *wt*, 82.06 ± 5.35; *him-6(ok412)*, 45.22 ± 12.54; *him-6^K275R^*, 76.78 ± 9.30], Supplementary Fig. S3E and F and Supplementary Table S4. The lack of asynapsis in late pachynema in *him-6^K275R^* indeed reinforces the idea that recombination is much less compromised in this genotype and that the few univalents detected in this mutant arise past a step in recombination, which would normally trigger asynapsis when it is compromised.

In summary, the overall numbers of MSH-5 and COSA-1 foci were lower in both mutants (*him-6(ok412)* and *him-6^K275R^*) than in the wild type, likely reflecting that a reduction in helicase-dependent D-loop ejection reduces the overall number of CO-eligible intermediates. However, the counts in mid-pachynema were more similar to the wild type in the *him-6^K275R^* mutant (Fig. 3B, and Supplementary Fig. S3C and D), suggesting that the helicase supports the recombination process with a structural role. Nevertheless, the helicase activity of HIM-6 is vital to generate wild type numbers of MSH-5 and COSA-1 foci (Fig. 3B, and Supplementary Fig. S3C and D). Importantly, helicase inactive *him-6* mutants process recombination intermediates more efficiently, as evidenced by the lack of elevated apoptotic corpses (Fig. 3A) and the lack of asynapsed chromosomes in late pachynema nuclei (Supplementary Fig. S3E and F, and Supplementary Table S4).

### HIM-6 helicase activity suppresses extra COs but predominant CO placement to chromosome arms does not require it

Mutants of the BTR complex members (*him-6(ok412)*, *rmh-1(jf54)*, *rmif-2(jf113)*, and *top-3-ZnF* mutants) display an altered distribution of COs compared with the wild type [5, 16, 21, 22]. In the wild type, there is usually one CO per connected bivalent chromosome pair, and COs are enriched on the chromosome arms but largely absent from chromosome centers. However, in this class of mutants, the opposite localization was observed: there was an increase in COs at chromosome centers and a dramatic decrease on chromosome arms. COs on chromosomes arms are thought to help to correctly define chromosome domains that are later important for chromosome segregation. Cohesion is opened during the first division on the short arm but it persists on the long arm. When COs occur at the center of a chromosome, the chromosome fails to exhibit the distinct short and long arms of the parental bivalents, resulting in improper segregation of the chromosome [58, 59]. Therefore, we next investigated the role of HIM-6 helicase activity in positioning COs.

In order to track the distribution of COs, we created a Hawaiian *C. elegans* strain containing the *him-6^K275R^* mutation and followed recombination in hybrid Bristol/Hawaii oocytes by tracking SNPs on chromosome V (Fig. 3C, for the crossing scheme, see Jagut *et al.*, 2016 [16]). For comparison, we analyzed the wild type and *him-6(ok412)* alleles in the same way. Surprisingly, the *him-6^K275R^* mutant had a wild type distribution of COs on chromosome arms (interval AB: 0.098 for *wt* versus 0.090 for *him-6^K275R^*; interval DE: 0.181 for *wt* versus 0.192 for *him-6^K275R^*), with additional COs present at the center of the chromosome (interval BC: 0.278 for *wt* versus 0.067 *him-6^K275R^*; interval CD: 0.014 for *wt*, versus 0.137 for *him-6^K275R^*) (Fig. 3C and D). The recombination frequency in the total sample population was compared between genotypes using Fisher’s exact test. The overall recombination frequency was elevated in *him-6^K275R^* (47.06%) compared with the wild type (30.23%, *P*= 0.0012), whereas the frequency in *him-6(ok412)* (31.52%) was comparable to the wild type (*P*= 0.8281). The recombination frequency in *him-6(ok412)* was significantly lower than in *him-6^K275R^* (*P*= 0.0012). The frequency of double CO events was comparable among all three genotypes (1.86% in *wt*, 2.72% in *him-6(ok412)*, 1.57% in *him-6^K275R^*, Fig. 3E). The lack of double COs in spite of having an increased recombination frequency in the *hd him-6* mutant suggests that more chromatids are involved in the exchange.

Taken together, these results suggest that HIM-6 helicase activity is dispensable for the proper placement of off-centered COs (unlike for all other mutants of BTR complex proteins). However, the total recombination frequency is elevated in *him-6^K275R^*, likely by introducing additional COs at chromosome centers and generating fewer nonexchange chromatids.

### Lack of HIM-6 protein and lack of HIM-6 helicase activity have different effects on dHJ conformation

**Figure 4.**
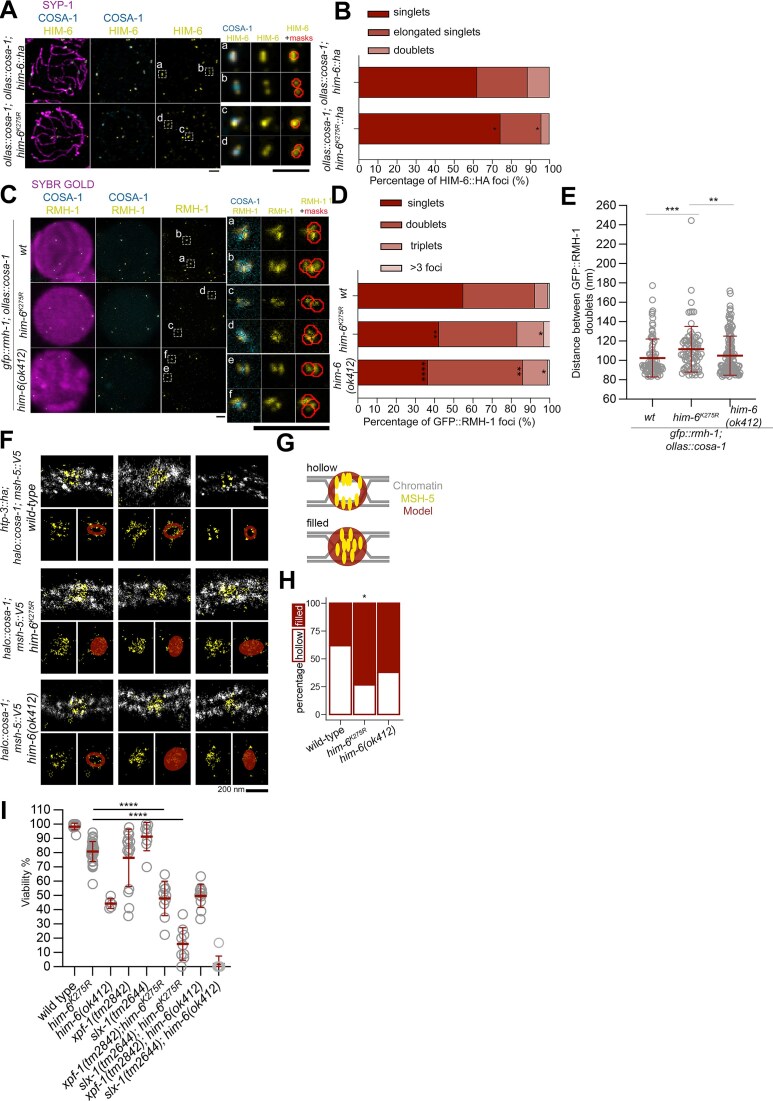
HIM-6 helicase activity shapes the geometry of the dHJ. (**A**) Left panel, fully Z-projected SIM images of meiotic spreads of the indicated genotypes stained to visualize SYP-1, OLLAS::COSA-1, and HIM-6::HA. The scale bars represent 1 μm. Insets an and c are representative examples of elongated singlets, and insets b and d of doublets. Enlarged images of insets a–d show the HA channel (yellow) to depict HIM-6::HA and OLLAS (cyan) to depict OLLAS::COSA-1 and masks obtained from the SpotMAX-based analysis are overlaid onto the HIM-6::HA channel for clarity. The scale bar represents 1 μm. (**B**) Graph showing the percentage of foci in each category (singlets, elongated foci, and doublets). A Fisher’s exact test was used to compare the frequency of occurrence of each focus type between genotypes ****P< 0.0001, ***P< 0.001, **P< 0.01, *P< 0.05, and not significant (ns), *P*≥ 0.05. The statistical significance (*P-*value) of any difference between the genotypes was: singlets, * (0.0142); elongated foci, ns (0.2753); and doublets * (0.0136). The percentage of each focus type in each genotype was as follows. *ollas::cosa-1; him-6::ha*: singlets, 62.02%; elongated singlets, 26.44%; and doublets, 11.54%. *ollas::cosa-1; him-6^K275R^::ha*: singlets, 74.23%; elongated singlets, 21.47%; and doublets, 4.29%. In *ollas::cosa-1; him-6::ha*, 208 putative CO sites were counted in 34 nuclei; and in *ollas::cosa-1; him-6^K275R^::ha* 163 CO sites were counted in 30 nuclei. (**C**) Left panel, STED images of meiotic spreads of the indicated genotypes stained to visualize the chromatin (stained using SYBR Gold), OLLAS::COSA-1, and GFP::RMH-1. The scale bars represent 1 μm. Insets an and c represent doublets, and insets b and d of triplets. Enlarged images of insets a–d show the GFP channel (yellow) to depict GFP::RMH-1 and OLLAS (cyan) to depict OLLAS::COSA-1 and masks obtained from the SpotMAX-based analysis are overlaid onto the GFP::RMH-1 channel for clarity. (**D**) Graph showing the percentage of foci in each category (singlets, doublets, triplets and > 3 foci) in the indicated genotypes. A Fisher’s exact test was used to compare the frequency of occurrence of each focus type between genotypes ****P< 0.0001, ***P< 0.001, **P< 0.01, *P< 0.05, and not significant (ns), *P*≥ 0.05. The percentage of each focus type in each genotype was as follows. *gfp::rmh-1; ollas::cosa-1 (wt)*: singlets, 54.94%; doublets, 37.34%; triplets, 6.87%; and > 3 foci, 0.86%. *gfp::rmh-1; ollas::cosa-1; him-6^K275R^::ha*: singlets, 42.29%; doublets, 40.80%; triplets, 13.93%; and > 3 foci, 2.99%. *gfp::rmh-1; ollas::cosa-1; him-6(ok412)*: singlets, 36.46%; doublets, 49.65%; triplets, 12.85%; and >3 foci, 1.04%. The *P*-values of the comparisons are given in the following order: *wt* versus *gfp::rmh-1; ollas::cosa-1; him-6^K275R^*, *wt* versus *gfp::rmh-1; ollas::cosa-1; him-6(ok412)* and *gfp::rmh-1; ollas::cosa-1; him-6^K275R^* versus *gfp::rmh-1; ollas::cosa-1; him-6(ok412)*. Singlets: **(0.0094), ****(<0.0001), and ns(0.2203). Doublets: ns(0.4902), **(0.0059), and ns(0.0650). Triplets: *(0.0169), *(0.0285), and ns(0.787). >3 foci: ns(0.1522), ns(>0.999), and ns(0.1704). (**E**) Graph indicating the distance between the GFP::RMH-1 doublets. The distance spanned by the doublets in nm (mean ± SD) for each genotype is as follows. *wt*, 102.5 ± 19.63; *gfp::rmh-1; ollas::cosa-1; him-6^K275R^*, 111.5 ± 23.56; and *gfp::rmh-1; ollas::cosa-1; him-6(ok412)*, 105 ± 20.14. The Mann–Whitney test was used to compare distances: ****P< 0.0001, ***P< 0.001, **P< 0.01, *P< 0.05, and not significant (ns), *P*≥ 0.05. The statistical significance (*P*-value) of differences in GFP::RMH-1 doublets distances is as follows: *wt* versus *gfp::rmh-1; ollas::cosa-1; him-6^K275R^*, ***(0.0006), wt versus *gfp::rmh-1; ollas::cosa-1; him-6(ok412)*, ns(0.482), and *gfp::rmh-1; ollas::cosa-1; him-6^K275R^* versus *gfp::rmh-1; ollas::cosa-1; him-6(ok412)*, **(0.0079). (**F**) Representative images of late MSH-5 foci in single-molecule localization micrographs. The chromosome axis (anti-HTP-3 antibody) is shown in gray, and MSH-5::V5 (anti-V5 antibody) is shown in yellow. The scale bar represents 200 nm. Red shapes represent elliptical shapes used to assess the structure. The Supplementary Videos S1–3 depict a 3D view of these structures. (**G**) Cartoons describing the different structures of individual foci: hollow elliptical model (top) and filled elliptical model (bottom). Models are shown as red ellipses, MSH-5 as yellow ellipses, and chromatin by gray lines. (**H**) Quantification of fit results for hollow and filled elliptical models. The number of foci analyzed per genotype (*n*) and the percentage of hollow foci for each genotype were: wild type (*halo::cosa-1; msh-5::V5*), *n* = 21, 61.9%; *halo::cosa-1; msh-5::V5 him-6^K275R^*, *n* = 15, 26.7%; and *halo::cosa-1; msh-5::V5 him-6(ok412)*, *n* = 37, 37.8%. The Fisher’s exact test was used to determine the statistical significance (*P* values) of differences in comparisons between genotypes: *wt* versus *him-6^K275R^*, * (0.049); *wt* versus *him-6(ok412)*, ns (0.10); and *him-6^K275R^* versus *him-6(ok412)*, ns (0.53). (**I**) The percentage viability of various genotypes. The viability counts of *him-6^K275R^* are identical to those shown in Fig. 1B. The number of animals (*n*) used and percentage viability (mean ± SD) for each genotype were: wild type (*wt*), *n* = 10, 98.29 ± 2.33; *him-6^K275R^*(as previously mentioned in Fig. 1B), *n* = 38, 80.77 ± 6.95; *him-6(ok412)*, *n* = 5, 44.31 ± 3.44; *xpf-1(tm2842)*, *n* = 15, 76.32 ± 20.12; *slx-1(tm2644)*, *n* = 8, 91.32 ± 9.95; *xpf-1(tm2842); him-6^K275R^*, *n* = 10, 47.92 ± 12.07; *slx-1(tm2644); him-6^K275R^*, *n* = 9, 15.93 ± 11.43; *xpf-1(tm2842); him-6(ok412)*, *n* = 11, 49.67 ± 8.11; and *slx-1(tm2644); him-6(ok412)*, *n* = 9, 1.85 ± 5.56. The Mann–Whitney test was used to compare percentage viability between mutants and the wild type ****P< 0.0001, ***P< 0.001, **P< 0.01, *P< 0.05, and not significant (ns), *P*≥ 0.05. The statistical significance (*P*-value) of all differences between the genotypes are in the Supplementary Table S5.

Recombination sites are marked by the cyclin-like protein COSA-1 [52]. The pro-CO factors HIM-6 and MSH-5 were previously shown to localize as an orthogonal doublet flanking a COSA-1 focus at presumptive CO sites [16, 31]. We investigated the localization pattern of HIM-6^K275R^::HA at late CO sites, where wild type HIM-6 forms a doublet signal. We found that similar doublets can, in principle, also form in the mutant and can be visualized by SIM (Fig. 4A). By quantifying foci shapes, we found a decreased percentage of doublets among all foci in *him*-*6^K275R^::ha* (4.29%) compared with the wild type (11.54%, Fig. 4B). A possible explanation is that the two signals are closer together and/or more spread out in *him*-*6^K275R^::ha* and are, therefore, detected as a single signal or an elongated focus.

RMH-1 is known to occur as doublets flanking COSA-1 foci in late-pachynema [16]. We employed STED microscopy to investigate the role of the helicase activity of HIM-6 in the positioning of RMH-1 foci (Fig. 4C and D). We found that GFP::RMH-1 in the *hd him-6* mutant appeared as doublets to a similar extent as in the wild type (37.34% in *wt* versus 40.8% in *him-6^K275R^*). There was an increased number of doublets observed in *him-6(ok412)*. Interestingly, we also observed triplets of GFP::RMH-1 which were seen more frequently in both the mutants when compared to the wild type (6.86% in *wt*, 13.93% in *him-6^K275R^* and 12.85% in *him-6(ok412)*). Moreover, the distance between the GFP::RMH-1 doublets in the *him-6^K275R^* mutant is significantly higher than in the wild type and in *him-6(ok412)* (Fig. 4E, mean ± SD in nm 102.5 ± 19.63 in *wt*, 111.5 ± 23.56 in *him-6^K275R^* and 105 ± 20.14 in *him-6(ok412)*).

To further explore the role of the HIM-6 helicase activity in meiosis, we closely investigated the structure of late recombination sites by mapping the 3D organization of MSH-5::V5 signal at late recombination intermediates using SMLM. Whereas HIM-6 foci can be readily classified into singlets, elongated foci, and doublets in SIM images of chromosome spreads (Fig. 4A), 3D-SMLM images revealed a surprisingly high degree of diversity in MSH-5::V5 foci across all genotypes (Fig. 4F), Supplementary Videos 1-3. Despite this, the MSH-5::V5 signal colocalized with both chromosome axes (highlighted with yellow) in most foci, which we interpreted as corresponding to dHJ doublets in chromosome spreads. In wild type animals, some MSH-5::V5 foci adopted an open conformation, resembling two lines or hollow rings. In contrast, in the *him-6^K275R^* mutant, the foci resembled a closed circle rather than a ring (Fig. 4G). To quantify these differences, we fitted the 3D localization of MSH-5::V5 foci to a model of a hollow elliptical ring or of a filled ellipse using LocMoFit [46] (Fig. 4 G and H). Comparison of the goodness-of-fit-ratios of filled and hollow models (Fig. 4H) showed that MSH-5::V5 foci in wild type late recombination sites are almost twice as likely to adopt a hollow conformation than in *hd* mutants (*wt halo::cosa-1; msh-5::v5*, 61.9%; *halo::cosa-1; msh-5::v5 him-6^K275R^*, 26.7%; *P-*value, 0.049), suggesting that HIM-6-mediated helicase activity is required to change dHJs to an open conformation. In contrast, MSH-5::V5 foci in the *him-6(ok412)* null mutant formed a more hollow structure than in *him-6^K275R^* but a more filled-in structure than in the wild type (*halo::cosa-1; msh-5::v5 him-6(ok412)*, 37.8% of foci are hollow; *P*-value when compared with *wt*, 0.10; *P-*value when compared with *him-6^K275R^*, 0.53). This suggests an intermediate phenotype for *him-6(ok412)* and that HIM-6 may have helicase-independent functions in regulating the dHJ structure.

Epistasis analysis of possible resolvase pathways suggests that final cleavage of the joint DNA molecules is mediated by two parallel redundant pathways (SLX-1/MUS-81 and XPF-1/HIM-6) [5, 8]. Therefore, we assessed whether hatching rates in the *xpf-1(tm2842); him-6^K275R^* double mutants are similarly nonadditive as has been seen for *xpf-1(tm2842); him-6(ok412)*, and whether *him-6^K275R^* is synthetic lethal with *slx-1(tm2644)* [5, 7, 8]. Surprisingly, we found that *him-6^K275R^* is synthetic lethal with either *slx-1(tm2644)* or *xpf-1(tm2842)* (Fig. 4I and Supplementary Table S5).

These combined results suggest a model in which during joint molecule resolution the accuracy of cutting by XPF-1 is supported by optimal presentation by the Bloom helicase, which might promote appropriate spacing between the two ends of the joint DNA molecules. The unexpected synthetic lethality of the double *him-6^K275R^* and *xpf-1(tm2842)* mutation may be caused by persistent residency of the catalytically inactive helicase preventing access of the alternative pathway nucleases to the structure.

## Conclusion

Studies in numerous organisms have established that Bloom orthologs are crucial for normal meiosis by mediating NCO and CO formation during recombination. The biochemical activities required to generate NCOs by unwinding recombination intermediates have been recapitulated in *in vitro* reconstitution experiments, and genetic analysis of meiotic mutants consistently detects supernumerary COs. Although the contribution of Bloom helicase to CO formation is less well understood, unified models suggest that its unwinding activities generate a continuous pool of substrates that can be selected to become a CO [26]. However, a more active contribution to generating COs by facilitating the formation of a specific joint molecule structure by either stabilizing strand-exchange intermediates or influencing, for example, the proper dimensions of the dHJ to enable concerted resolvase cuts remains speculative [60–62]. *In vitro* reconstitution experiments of yeast dHJ resolution also suggest that Sgs1, the yeast Bloom ortholog, might contribute a necessary junction migration activity [62]. However, *in vivo* evidence is lacking for this activity.

Our study of catalytically inactive HIM-6 suggests that HIM-6 contributes to successful meiosis by, first, providing a continuous flux of substrates for CO formation, likely via its unwinding activities. In both null and catalytically inactive mutants, the number of MSH-5- and COSA-1-decorated intermediates in early pachynema were reduced, although sufficient intermediates were available to make COs (Fig. 3B and E and Supplementary Fig. S3C and D). Moreover, both the null and catalytically inactive mutants are synthetic lethal with *smc-5* mutants, confirming their lack of DNA unwinding activity (Fig. 1E) [63].

Second, a structural role for HIM-6 is sufficient to channel CO intermediates into the preferred pathway to generate COs on the terminal third of chromosome arms: wild type CO numbers on chromosome arms were detected in *hd him-6* mutants; in contrast, the number of COs on one of the chromosome arms (interval DE) was markedly reduced in the null mutant (Fig. 3C and D). The structural support from HIM-6 to generate wild type COs is also reflected in the markedly improved egg hatching rates and a significant reduction of univalents in diakinesis nuclei in *hd him-6* mutants (Fig. 1B and D), increased MSH-5 and COSA-1 foci in zones 2 and 3 in *hd* mutants compared with the null mutant (Fig. 3B, and Supplementary Fig. S3C and D), and reduced apoptosis in *hd* versus null mutant (Fig. 3A). Finally, and most excitingly, we provide evidence that HIM-6 catalytic activity influences the geometry of the joint DNA molecules. Location of the MutSγ component MSH-5 was more restricted in *hd him-6* mutants and less likely to form the ring-like structure observed in the wild type (Fig. 4F–H and Supplementary Videos 1-3). It is tempting to speculate that restricted MSH-5 localization in the mutant might result from aberrant HIM-6-mediated branch migration and that altered geometry of the joint DNA molecules impedes the efficiency of directed cutting by XPF-1 nuclease to generate CO-biased cleavage products which results in the formation of univalents (Fig. 1D). The lack of the helicase activity in the *hd him-6* mutant alters the spacing of RMH-1 foci (Fig. 4E) further supporting this model. This model was also supported by epistasis experiments that showed synthetic lethality between *hd him-6* and the *slx-1* or *xpf-1* mutants (Fig. 4I), in contrast to the lack of synthetic lethality reported for double mutants of *xpf-1* with the *him-6* null mutants [5, 8] (Fig. 4I and Supplementary Table S5). Our results showing MSH-5 distribution on putative dHJs in the *him-6* null mutant hint that HIM-6 may be involved in stabilizing joint DNA molecule geometry by ensuring optimal spacing (Fig. 4F–H).

Our combined results are compatible with a model in which Bloom helicase HIM-6 promotes CO formation during meiotic recombination in at least three ways through both structural and catalytic activities. We propose that HIM-6 helicase activity shapes the geometry of the dHJ to promote efficient biased resolution of the structure.

## Supplementary Material

gkaf1030_Supplemental_Files

## Data Availability

The data underlying this article are available in the article and in its online supplementary material. The data underlying this article are available in the article and in its online supplementary material.

## Notes

Present address: Stanford University, Department of Developmental Biology, 94305-5329 Stanford, CA, United States

Present address: Institute for Research in Biomedicine, Università della Svizzera italiana, Faculty of Biomedical Sciences, 6500 Bellinzona, Switzerland
