# Supplementary material for: Bloom helicase contributes to successful crossover formation with both catalytic and structural roles in Caenorhabditis elegans meiosis
Source: Nucleic Acids Res. 2025 Oct 22;53(19):gkaf1030. doi: 10.1093/nar/gkaf1030 (PMC12541369; doi:10.1093/nar/gkaf1030)
Supplement: gkaf1030_Supplemental_Files [file gkaf1030_supplemental_files.zip › Supplementary Materials_revised_02.10.2025.pdf]

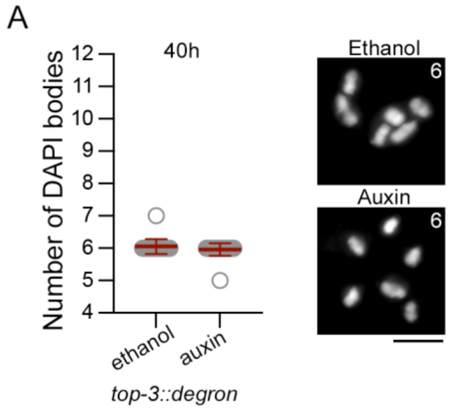

Supplementary Figure 1

(**A**) Left panel, TOP-3 was depleted using an AID degron-based system, as used previously (1,2). The control condition was the solvent for auxin (labeled ethanol). The number of DAPI bodies counted in the -1 oocyte after 40 hours of depletion is shown. The number of oocytes counted (n) and number of DAPI bodies (mean ± SD) were: ethanol, n=19, 6.05±0.23; and auxin, n=26, 5.96±0.20. A Mann–Whitney test showed no statistical difference in DAPI body numbers between treatments (p=0.499). Right panel, representative images of -1 oocytes in diakinesis, with full projection of the images. The number of DAPI bodies counted in each example is shown. The scale bar represents 5 µm.

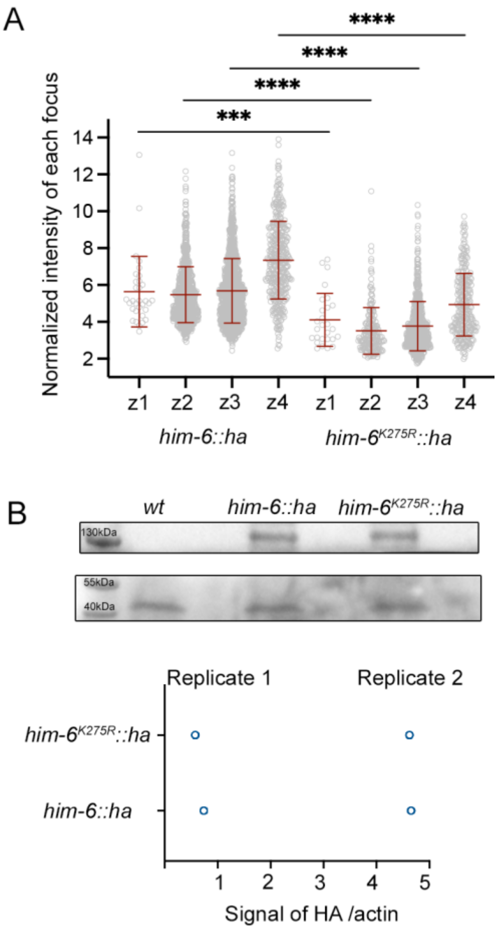

Supplementary Figure 2

(**A**) The graph shows the normalized intensity of HA foci in the wild type and mutant. The number of nuclei measured in each zone (n) and the intensity of foci (mean ± SD) were as follows. *him-6::ha*: zone 1, n=32, 5.64±1.91; zone 2, n=820, 5.48±1.52; zone 3, n=1281, 5.69±1.75; and zone 4, n=399, 7.34±2.11. *him-6^{K275R}::ha*: zone 1, n=27, 4.11±1.44; zone 2, n=205, 3.51±1.37; zone 3, n=696, 3.77±1.34; and zone 4, n=278, 4.94±1.70. The Mann–Whitney test was used to compare foci numbers in the mutant and wild type (*him-6::ha*): ****, p<0.0001; ***, p<0.001; **, p<0.01; *, p<0.05; and not significant (ns), p≥0.05. The statistical significance (p value) of differences in HA foci numbers between *wt* and *him-6^{K275R}::ha* were: zone 1, *** (0.0002); zone 2, **** (<0.0001); zone 3, **** (<0.0001); and zone 4, **** (<0.0001).

(**B**) Upper panel, western blot analysis of the HA signal in the *him-6::ha* (wild type) and *him-6^{K275R}::ha* strains, with actin used to normalize protein levels. Lower panel, graph showing the normalized HA signals (HA signal intensity/actin signal intensity) in two replicate samples of *him-6::ha* and *him-6^{K275R}::ha*.

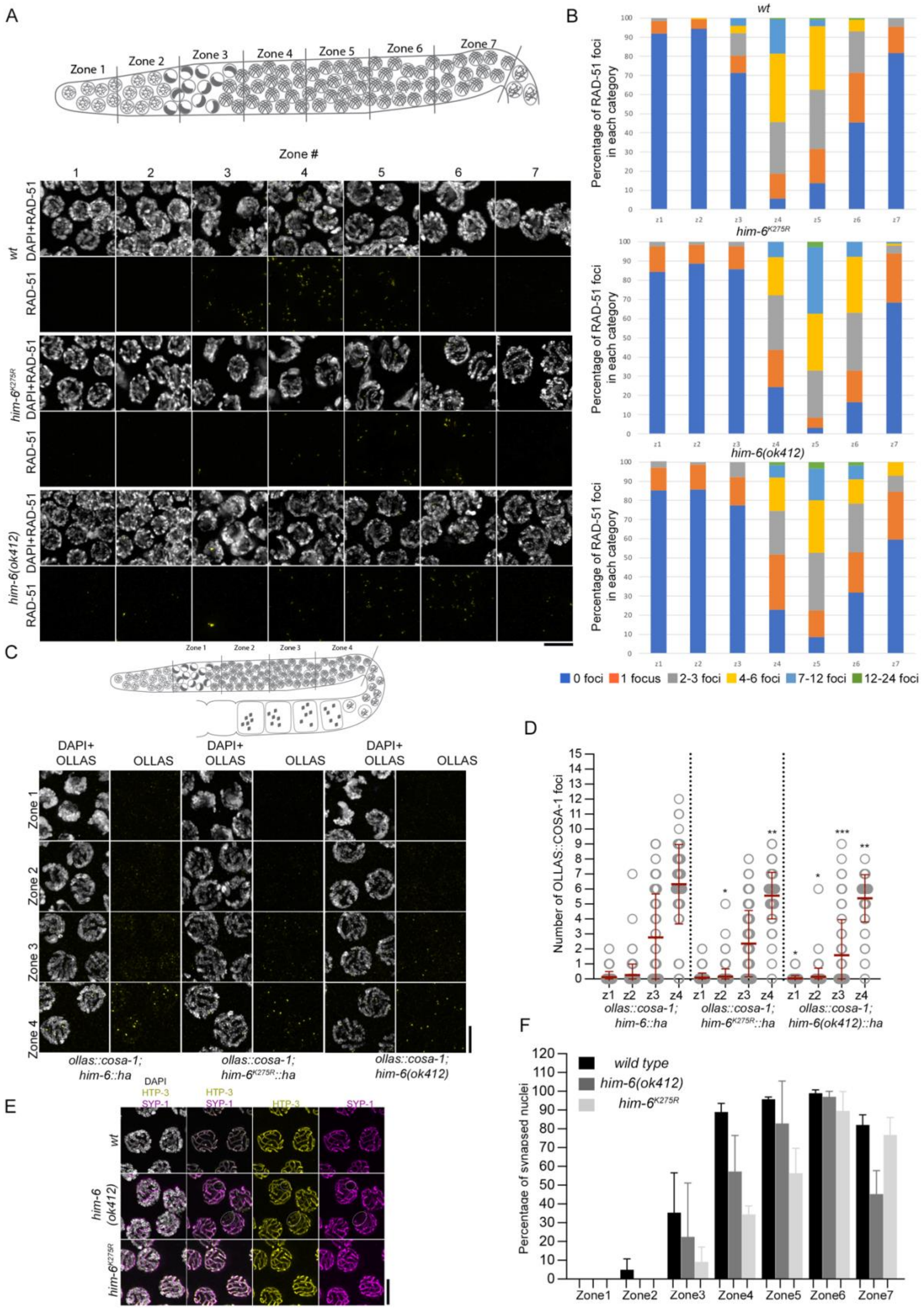
A
Zone 1
Zone 2
Zone 3
Zone 4
Zone 5
Zone 6
Zone 7
Zone #
1 2 3 4 5 6 7
wt DAPI+RAD-51
RAD-51
him-6K275R DAPI+RAD-51
RAD-51
him-6(ok412) DAPI+RAD-51
RAD-51
B
wt
him-6K275R
him-6(ok412)
Percentage of RAD-51 foci in each category
z1 z2 z3 z4 z5 z6 z7
0 foci
1 focus
2-3 foci
4-6 foci
7-12 foci
12-24 foci
C
Zone 1
Zone 2
Zone 3
Zone 4
DAPI+ OLLAS
OLLAS
ollas::cosa-1; him-6::ha
ollas::cosa-1; him-6K275R::ha
ollas::cosa-1; him-6(ok412)
D
Number of OLLAS::COSA-1 foci
z1 z2 z3 z4
ollas::cosa-1; him-6::ha
ollas::cosa-1; him-6K275R::ha
ollas::cosa-1; him-6(ok412)::ha
E
DAPI
HTP-3
SYP-1
wt
him-6 (ok412)
him-6K275R
F
wild type
him-6(ok412)
him-6K275R
Percentage of synapsed nuclei
Zone1
Zone2
Zone3
Zone4
Zone5
Zone6
Zone7

Supplementary Figure 3

(**A**) Cartoon illustrating division of the gonad into seven equal zones from the distal tip to late pachynema. Lower left panel, representative images of RAD-51 staining in wild type (*wt)*, *him-6*^{K275R}, and *him-6(ok412)* gonads. The scale bar represents 5 µm. RAD-51 foci appeared and disappeared slightly later in the mutants, which led to a shift in the maximum number of foci per zone from zone 4 (as in the *wt*) to zone 5.

(**B**) Graphs with colored bars indicating the percentage of nuclei that contain no RAD-51 foci, 1 focus, 2–3 foci, 4–6 foci, 7–12 foci, and >24 foci. Fisher's exact t test was used to compare differences in RAD-51 foci numbers in each zone between genotypes. Detailed results of the analysis are shown in Supplementary Table 3. The number of nuclei per zone assessed in each genotype were as follows. *wt*: zone 1, 123; zone 2, 122; zone 3, 101; zone 4, 140; zone 5, 117; zone 6, 101; and zone 7, 65. *him-6*^{K275R}: zone 1, 57; zone 2, 201; zone 3, 195; zone 4, 172; zone 5, 152; zone 6, 127; and zone 7, 101. *him-6(ok412)*: zone 1, 140; zone 2, 139; zone 3, 128; zone 4, 102; zone 5, 116; zone 6, 110; and zone 7, 84.

(**C**) Schematic representation of the four zones defined on the germline, from meiotic entry to late pachynema. Bottom panel, representative images of germline nuclei stained with anti-OLLAS and counterstained with DAPI of the genotypes *ollas::cosa-1; him-6::ha*, *ollas::cosa-1; him-6*^{K275R}*::ha*, and *ollas::cosa-1; him-6(ok412)* from zone 1 (meiotic entry) to zone 4 (late pachynema). Zones are defined in Figure 2A. The scale bar represents 5 µm.

(**D**) Graph showing number of OLLAS::COSA-1 foci in the indicated genotypes. The number of nuclei assessed in each zone (n) and number of OLLAS::COSA-1 foci (mean ± SD) were as follows. *ollas::cosa-1; him-6::ha*: zone 1, n=134, 0.12±0.37; zone 2, n=178, 0.26±0.74; zone 3, n=132, 2.77±2.89; and zone 4, n=57, 6.32±2.65. *ollas::cosa-1; him-6*^{K275R}*::ha*: zone 1, n=304, 0.09±0.31; zone 2, n=308, 0.16±0.51; zone 3, n=244, 2.35±2.21; and zone 4, n=139, 5.55±1.55. *ollas::cosa-1; him-6(ok412)*: zone 1, n=175, 0.03±0.18; zone 2, n=159, 0.14±0.58; zone 3, n=111, 1.58±2.38; and zone 4, n=81, 5.38±1.58. The Mann–Whitney test was used to compare foci numbers in zone 1–4 of mutant and wild type (*ollas::cosa-1;him-6::ha*) strains: ****, p<0.0001; ***, p<0.001; **, p<0.01; *, p<0.05; and not significant (ns), p≥0.05. The statistical significance (p value) of differences in OLLAS::COSA-1 foci number for each zone for all comparisons is given in the following order: *ollas::cosa-1; him-6::ha* vs *ollas::cosa-1;him-6*^{K275R}*::ha*; *ollas::cosa-1; him-6::ha* vs *ollas::cosa-1;him-6(ok412)*; and *ollas::cosa-1;him-6*^{K275R}*::ha* vs *ollas::cosa-1;him-6(ok412)*. Zone 1: ns (0.3171), * (0.0125), and ns (0.0658). Zone 2: * (0.0319), * (0.0178), and ns (0.5132). Zone 3: ns (0.4508), *** (0.0008), and ** (0.0011). Zone 4, ** (0.0012), ** (0.0011), and ns (0.9783).

(**E**) Representative images from zone 7 of HTP-3 and SYP-1 stained in gonads of *wt*, *him-6(ok412)* and *him-6*^{K275R}. As in (A), the gonad was divided into seven equal zones from the distal tip to late pachynema. The asynapsed regions in the nucleus are highlighted by the dotted ellipses. The scale bar represents 5µm.

(**F**) Graph shows the percentage of synapsed nuclei in the total number of nuclei for each of the seven zones in the indicated genotypes. Fisher's exact test was employed to test for

statistical significance. Results of this analysis are shown in Supplementary table 4. The number of nuclei per zone assessed in each genotype were as follows. *wt*: zone 1, 109; zone 2, 137; zone 3, 95; zone 4, 92; zone 5, 91; zone 6, 77; and zone 7, 54. *him-6*^{K275R}: zone 1, 105; zone 2, 109; zone 3, 84; zone 4, 70; zone 5, 86; zone 6, 92; and zone 7, 65. *him-6(ok412)*: zone 1, 114; zone 2, 130; zone 3, 109; zone 4, 142; zone 5, 109; zone 6, 98; and zone 7, 76.

Supplementary table S1. List of strains used in this study

C. elegans strains	Source	strain code
wild type (N2 Bristol)	https://cgc.umn.edu/strain/search	
Hawaiian	https://cgc.umn.edu/strain/search	CB4856
him-6^{K275R}/nT1 (IV;V)	this study	UV287
him-6^{K275R}::ha/nT1 (IV;V)	this study	UV288
him-6^{K275A}::ha/nT1 (IV;V)	this study	UV290
him-6(ok412)	(3)	VC193
him-6(ok412) in the Hawaiian background<br>*him-6(syb9242)*	Suny Biotech	PHX9242
him-6^{K275R} in the Hawaiian background	this study	UV289
him-6(ok412) gfp::msh-5/nT1 (IV;V)	this study	UV292
him-6^{K275R} gfp::msh-5/nT1 (IV;V)	this study	UV293
gfp::msh-5	(4)	NSV129
ollas::cosa-1 III; him-6::ha IV	this study	UV294
ollas::cosa-1 III; him-6^{K275R} IV/nT1 (IV;V)	this study	UV295
ollas::cosa-1 III; him-6(ok412) IV/nT1 (IV;V)	this study	UV296
M01E11.3(jf92[M01E11.3::unc-119(+)]) I; jfsi38 [gfp::rmh-1 ; cb-unc-119(+)] II	(5)	UV302
M01E11.3(jf92[M01E11.3::unc-119(+)]) I; jfsi38 [gfp::rmh-1 ; cb-unc-119(+)] II; him-6(ok412)/nT1 IV (IV;V)	this study	UV298

jfsi38 [gfp::rmh-1 ; cb-unc-119(+)] II; him-6(ok412)/nT1 (IV;V)	(5)	UV299
M01E11.3(jf92[M01E11.3::unc-119(+)]) I; jfsi38 [gfp::rmh-1 ; cb-unc-119(+)] II; him-6^{K275R}::ha/nT1 (IV;V)	this study	UV297
slx-1(tm2644) I	National Bioresource Project for the Nematode, Japan	TG1868
xpf-1(tm2842) II	National Bioresource Project for the Nematode, Japan	TG1660
slx-1(tm2644) I ; him-6^{K275R}/nT1 (IV;V)	this study	UV300
xpf-1(tm2842) II ; him-6(ok412)	(6)	TG2455
*slx-1(tm2644) I ; him-*6(ok412)*/nT1 (IV;V)*	this study	UV329
xpf-1(tm2842) II; him-6^{K275R}/nT1 (IV;V)	this study	UV301
halo::cosa-1(ske25) III ; msh-5::V5(ske49) him-6(ok412) IV/nT1 [qIs51] (IV;V)	this study	UV284
htp-3::ha (ske16-1) I; halo::cosa-1 (ske25) III; msh-5::V5(ske49) (IV)	Čavka *et al.*, in preparation	SMN311
halo::cosa-1 (ske25) III; msh-5::v5(ske49.2) him-6^{K275R}/nT1 (IV;V)	this study	SMN551
smc-5(ok2421)II; him-6(jf209)/nT1 (IV;V)	this study	UV291
smc-5(ok2421))/mIn1[dpy-10(e128) mIs14[myo-2::gpf; pes-10::gfp]] II	OMRF Knockout group	YE57
rmh-1[syb9786(GFP::rmh-1)] I	(7)	NSV688
gfp::rmh-1(syb9786) I; ollas::cosa-1 III	this study	UV327

gfp::rmh-1(syb9786) I; ollas::cosa-1 III; him-6(jf209) IV	this study	UV328
gfp::rmh-1(syb9786) I; ollas::cosa-1 III; him-6(ok412) IV	this study	UV326

Supplementary table S2. List of antibodies used

Antibodies	Source	Animal source	Final concentration	Preabsorbed
HA	Sigma H6908	rabbit	1:100	yes
OLLAS	ThermoFisher MA5-16125)	rat	1:500	Yes
GFP	Roche #11814460001	mouse	1:500	yes
RAD-51	Gift from Monique Zetka	rabbit	1:1000	
SYP-1	Gift from Enrique Martinez-Perez	chicken	1:400	
SYP-1	Gift from Nicola Silva	rabbit	1:1000	
HA	Covance catalog # MMS-101P	mouse	1:100	yes
OLLAS	GenScript, A01658	rabbit	1:1000	yes
HTP-3	(8)	chicken	1:200	
HTP-3	Gift from Yumi Kim	guinea-pig	1:500	
V5	Thermo Fisher Scientific, R960-25	mouse(monoclonal)	1:200	
HA (for Western blotting)	Sigma H6908	rabbit	1:3000	
actin (for Western blotting)	Santa Cruz Biotechnology #E1415	goat	1:3000	

anti-goat IgG-HRP (for Western blotting)	Santa Cruz Biotechnology (sc-2020)	donkey	1:8000	
anti-rabbit IgG-HRP (for Western blotting)	Pierce #31460	goat	1:25000	
anti-rabbit	Invitrogen A11034	goat	1:500	
anti-rat	Invitrogen A11077	goat	1:500	
anti-mouse	Invitrogen A11001	goat	1:500	
anti-rabbit	Invitrogen A11036	goat	1:500	
Anti-guinea pig	Invitrogen A11073	goat	1:500	
anti-mouse	Biotium 20014	donkey	1:500	
anti-chicken	Invitrogen A-21449	goat	1:250	
anti-mouse conjugated with AlexaFluor 647	Jackson Immunoresearch, AB_2340761	donkey(polyclonal)	1:50	
anti-chicken conjugated with CF660C	Jackson Immunoresearch, AB_2340347	donkey(polyclonal)	1:50	
anti-mouse STAR RED	Abberior (STRED-1001-500UG)	goat	1:100	
anti-rabbit STAR ORANGE	Abberior (STORANGE-1002-500UG)	goat	1:100	

Supplementary Videos:

Video 1- 3D representative view of SMLM images showing MSH-5::V5 and HTP-3 in the wild type.

Video 2- 3D representative view of SMLM images showing MSH-5::V5 and HTP-3 in *him-6*^{K275R}.

Video 3- 3D representative view of SMLM images showing MSH-5::V5 and HTP-3 in *him-6(ok412).*

Supplementary References

1. Dello Stritto, M.R., Bauer, B., Barraud, P. and Jantsch, V. (2021) DNA topoisomerase 3 is required for efficient germ cell quality control. *J Cell Biol*, **220**.
2. Zhang, L., Ward, J.D., Cheng, Z. and Dernburg, A.F. (2015) The auxin-inducible degradation (AID) system enables versatile conditional protein depletion in C. elegans. *Development*, **142**, 4374-4384.
3. Wicky, C., Alpi, A., Passannante, M., Rose, A., Gartner, A. and Muller, F. (2004) Multiple genetic pathways involving the Caenorhabditis elegans Bloom's syndrome genes him-6, rad-51, and top-3 are needed to maintain genome stability in the germ line. *Mol Cell Biol*, **24**, 5016-5027.
4. Janisiw, E., Dello Stritto, M.R., Jantsch, V. and Silva, N. (2018) BRCA1-BARD1 associate with the synaptonemal complex and pro-crossover factors and influence RAD-51 dynamics during Caenorhabditis elegans meiosis. *PLoS Genet*, **14**, e1007653.
5. Jagut, M., Hamminger, P., Woglar, A., Millonigg, S., Paulin, L., Mikl, M., Dello Stritto, M.R., Tang, L., Habacher, C., Tam, A. *et al.* (2016) Separable Roles for a Caenorhabditis elegans RMI1 Homolog in Promoting and Antagonizing Meiotic Crossovers Ensure Faithful Chromosome Inheritance. *PLoS Biol*, **14**, e1002412.
6. Agostinho, A., Meier, B., Sonneville, R., Jagut, M., Woglar, A., Blow, J., Jantsch, V. and Gartner, A. (2013) Combinatorial regulation of meiotic holliday junction resolution in C. elegans by HIM-6 (BLM) helicase, SLX-4, and the SLX-1, MUS-81 and XPF-1 nucleases. *PLoS genetics*, **9**, e1003591.
7. Engebrecht, J., Calidas, A., Li, Q., Ruiz, A., Padture, P., Barroso, C., Martinez-Perez, E. and Silva, N. (2025) Loss of Meiotic Double Strand Breaks Triggers Recruitment of Recombination-independent Pro-crossover Factors in C. elegans Spermatogenesis. *bioRxiv*, 2025.2006.2010.658785.
8. MacQueen, A.J., Phillips, C.M., Bhalla, N., Weiser, P., Villeneuve, A.M. and Dernburg, A.F. (2005) Chromosome sites play dual roles to establish homologous synapsis during meiosis in C. elegans. *Cell*, **123**, 1037-1050.
